# Comprehensive Analysis of the Triterpenoid Saponins Biosynthetic Pathway in *Anemone flaccida* by Transcriptome and Proteome Profiling

**DOI:** 10.3389/fpls.2016.01094

**Published:** 2016-07-25

**Authors:** Chuansong Zhan, Xiaohua Li, Zeying Zhao, Tewu Yang, Xuekui Wang, Biaobiao Luo, Qiyun Zhang, Yanru Hu, Xuebo Hu

**Affiliations:** ^1^Department of Medicinal Plant, College of Plant Science and Technology, Huazhong Agricultural UniversityWuhan, China; ^2^Center for Plant Functional Components, Huazhong Agricultural UniversityWuhan, China; ^3^National and Local Joint Engineering Research Center (Hubei) for Medicinal Plant Breeding and CultivationWuhan, China; ^4^The Hubei Provincial Engineering Research Center for Medicinal PlantsWuhan, China

**Keywords:** *Anemone flaccida*, triterpenoid saponin biosynthesis, transcriptome proteome, cytochrome P450, glycosyltransferase

## Abstract

**Background:** *Anemone flaccida* Fr. Shmidt (Ranunculaceae), commonly known as ‘Di Wu’ in China, is a perennial herb with limited distribution. The rhizome of *A. flaccida* has long been used to treat arthritis as a tradition in China. Studies disclosed that the plant contains a rich source of triterpenoid saponins. However, little is known about triterpenoid saponins biosynthesis in *A. flaccida*.

**Results:** In this study, we conducted the tandem transcriptome and proteome profiling of a non-model medicinal plant, *A. flaccida*. Using Illumina HiSeq 2000 sequencing and iTRAQ technique, a total of 46,962 high-quality unigenes were obtained with an average sequence length of 1,310 bp, along with 1473 unique proteins from *A. flaccida*. Among the *A. flaccida* transcripts, 36,617 (77.97%) showed significant similarity (*E*-value < 1*e*^-5^) to the known proteins in the public database. Of the total 46,962 unigenes, 36,617 open reading frame (ORFs) were predicted. By the fragments per kilobases per million reads (FPKM) statistics, 14,004 isoforms/unigenes were found to be upregulated, and 14,090 isoforms/unigenes were down-regulated in the rhizomes as compared to those in the leaves. Based on the bioinformatics analysis, all possible enzymes involved in the triterpenoid saponins biosynthetic pathway of *A*. *flaccida* were identified, including cytosolic mevalonate pathway (MVA) and the plastidial methylerythritol pathway (MEP). Additionally, a total of 126 putative cytochrome P450 (CYP450) and 32 putative UDP glycosyltransferases were selected as the candidates of triterpenoid saponins modifiers. Among them, four of them were annotated as the gene of CYP716A subfamily, the key enzyme in the oleanane-type triterpenoid saponins biosynthetic pathway. Furthermore, based on RNA-Seq and proteome analysis, as well as quantitative RT-PCR verification, the expression level of gene and protein committed to triterpenoids biosynthesis in the leaf versus the rhizome was compared.

**Conclusion:** A combination of the *de novo* transcriptome and proteome profiling based on the Illumina HiSeq 2000 sequencing platform and iTRAQ technique was shown to be a powerful method for the discovery of candidate genes, which encoded enzymes that were responsible for the biosynthesis of novel secondary metabolites in a non-model plant. The transcriptome data of our study provides a very important resource for the understanding of the triterpenoid saponins biosynthesis of *A. flaccida*.

## Introduction

**FIGURE 1:**
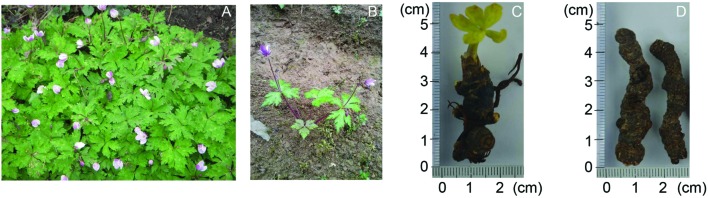
**The pictures of the medical plant, *Anemone flaccida*.** Wild *A. flaccida* grows in bunches where photo was taken in Tonggubao Village, Machi Town, Changyang County, Hubei Province, China **(A)**. A 3-year-old bush of *A. flaccida* was dispatched in a field for growing **(B)**. The germination of a 3-year-old *A. flaccida* in a conditioned chamber **(C)**. *A. flaccida* rhizomes of 5 or 6 years old were dried for medical uses **(D)**.

*Anemone flaccida* Fr. Shmidt (Ranunculaceae), commonly known as “Di Wu” in China (**Figures 1A–D**), is a perennial herb that is distributed around the world. In southern and central China, more than 50 species of *Anemone* has been investigated (Qin-Er, 2002; Marguerat and Baehler, 2010). Traditionally, the rhizome of *A*. *flaccida* is considered as a valuable Chinese traditional medicine for treatment of punch injury and rheumatoid arthritis (RA; Liu et al., 2015). Previous studies have shown that triterpenoid saponins are the main active ingredients in *Anemone* (Chen et al., 2009). Modern pharmacological research also elucidated that these kind of triterpenoid saponins had multiple therapeutic activities, including anti-tumor (Zhang et al., 2008), anti-inflammatory (Huang et al., 2014) anti-rheumatic (College, 1977), and anti-convulsant (Han et al., 2013). The total saponin fraction of the rhizome of *A. flaccida* is undergoing phase III clinical trials in China for the treatment of RA due to its significant anti-rheumatic, anti-inflammatory, and immunomodulatory effects *in vivo* (Huang et al., 2014). The extensive chemical and pharmacological studies on *A*. *flaccida* have paved a way for the further application of the medicine. However, the application of *Anemone* is largely hampered because of the short supply of the herbal materials. *A*. *flaccida* grows extremely slow with an active growth period around 2 months per year. It is usually distributed in mountains with the latitude above 1800 m (**Figures 1A–D**). Due to the slow growth and over-harvesting, *A*. *flaccida* population in some traditional production area, such as the southwest of Hubei province in China, is endangered. It is expected that the *A*. *flaccida* population in most area of Chinese territory will be threatened if the ongoing phase III clinical trial is approved. Understanding of the biosynthesis of active components of *A*. *flaccida* in molecular level may help us effectively propagate the germplasm and cultivation or even synthesis of the compound in other model species. Therefore, it is essential to clarify genes involved in the biosynthesis of *A. flaccida* triterpenoid saponins, and a thorough annotation of the enzymes in triterpenoid saponins biosynthetic pathway becomes necessarily.

**FIGURE 2:**
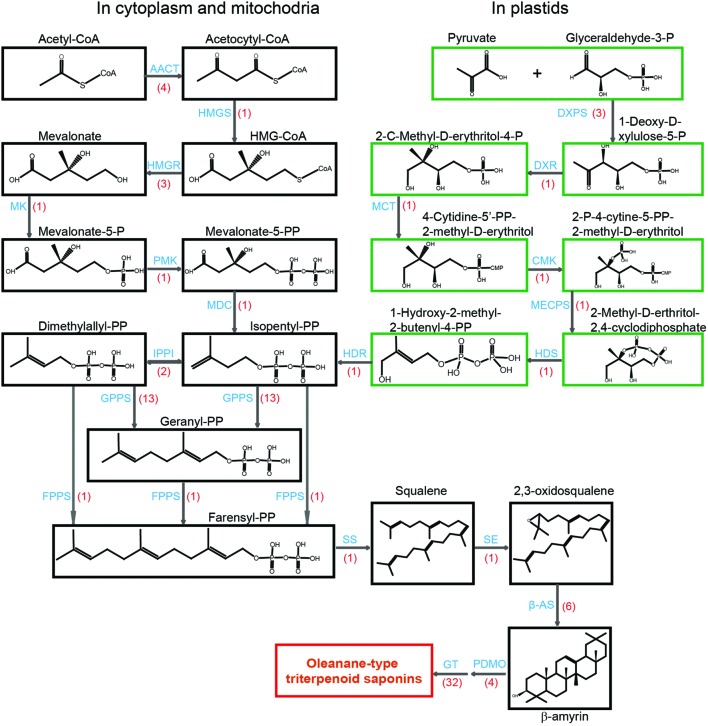
**Putative structural genes involved in the biosynthesis of terpenoid backbone in *Anemone flaccida*.** A flow diagram of biosynthetic pathway of triterpenoid in *A. flaccida*. The chemicals in the MVA pathway are shown in dark boxes, while green boxes are for MEP pathway. The words in blue are enzymes catalyze the reaction. The transcript numbers are shown in red brackets. Abbreviations used are: AACT, acetyl CoA *C*-acetyltransferase (*EC* 2.3.1.9); HMGS, 3-hydroxy-3-methylglutaryl CoA synthase (*EC* 4.1.3.5); HMGR, 3-hydroxy-3-methylglutaryl CoA reductase (*EC* 1.1.1.88); MK, mevalonate kinase (*EC* 2.7.1.36); PMK, phosphomevalonate kinase (*EC* 2.7.4.2); MDC, mevalonate-5-pyrophosphate decarboxylase (*EC* 4.1.1.33); IPPI, isopentenyl diphosphate isomerase (*EC* 5.3.3.2); GPPS, geranyl diphosphate synthase (*EC* 2.5.1.1); FPPS, farnesyl diphosphate synthase (*EC* 2.5.1.10); SS, squalene synthase (*EC* 2.5.1.21); SE, squalene epoxidase (*EC* 1.14.99.7); β-AS, β-amyrin synthase (*EC* 5.4.99.–); PDMO, cytochrome P450-dependent monooxygenases; GT, glycosyltransferases; DXPS, 1-deoxy-D-xylulose-5-phosphate synthase (*EC* 2.2.1.7); DXR, 1-deoxy-D-xylulose-5-phosphate reductoisomerase (*EC* 1.1.1.267); MCT, 2-*C*-methyl-D-erythritol 4-phosphate cytidylyl transferase (*EC* 2.7.7.60); CMK, 4-(cytidine 5’-diphospho)-2-*C*-methyl-D-erythritol kinase (*EC* 2.7.1.148); MECPS, 2-*C*-methyl-D-erythritol-2, 4-cyclodiphosphate synthase (*EC* 4.6.1.12); HDS, 4-hydroxy-3-methyl but-2-(*E*)-enyl diphosphate synthase (*EC* 1.17.7.1); HDR, 4-hydroxy-3-methyl but-2-(*E*)-enyl diphosphate reductase (*EC* 1.17.1.2).

The biosynthetic pathway of triterpenoid saponins in higher plants is located in different subcellular compartments. It has been established that triterpenoid saponins are synthesized by the isoprenoid pathway, and the active C5-unit isopentenyl pyrophosphate (IPP) is the precursor of all isoprenoids (Zhao et al., 2010). IPP is synthesized by either the mevalonate pathway (MVA) in cytoplasm and mitochondria or by the 2-*C*-methyl-D-erythrirtol-4-phosphate (MEP) pathway (**Figure 2**). Analysis of volatiles from the plant and it revealed that there is cross-talk between cytosolic and plastidial pathways of isoprenoid biosynthesis (Laule et al., 2003; Bartram et al., 2006). Over the past years, the next-generation sequencing technologies have revolutionized the analysis of genomic information (Nagalakshmi et al., 2010). The technique has been a powerful method for identifying candidate genes encoding enzymes responsible for the biosynthesis of secondary metabolites in the non-model plant (Asmann et al., 2008; Marguerat et al., 2008; Marguerat and Baehler, 2010; Tang et al., 2011; Mutasa-Göttgens et al., 2012; Zhao et al., 2013; Li et al., 2014). This technology had also been used in transcriptome profiling studies for medicinal plants, such as the triterpene biosynthesis of Luohanguo (*Siraitia grosvenorii*) and North American ginseng (*Panax quinquefolius*; Tang et al., 2011; Wu et al., 2013). Proteomics is an integrated analysis of protein structure, function and quantification in a given tissue or organ. Many new technologies have also been widely used in this area. Isobaric tags for relative and absolute quantification (iTRAQ) is a chemical labeling method that produces protein identification from peptide fragments and quantification from low mass reporter ions at the tandem mass spectrometry (MS/MS) level (Dean et al., 2007; Liu et al., 2013; Zhao et al., 2015). The new techniques make it possible for us to identify protein dynamic profiles in any complex biological process with high sensitivity and accuracy.

In the present study, a cDNA library generated from equal amount of RNA taken from the rhizomes and leaves of *A. flaccida* were sequenced using Illumina HiSeq 2000 sequencing platform. As a result, a total of 14.5 Gb valid data were obtained. We have confirmed that the total amounts of triterpenoid saponins in the rhizome were much higher than that in aerial parts of the plant (Zhao et al., unpublished data). Given these facts, the gene and protein expression profiles between the rhizomes and leaves of *A. flaccida*, were compared. To our knowledge, this study is the first exploration of the genes involved in the triterpenoid saponins biosynthetic pathway in *A. flaccida* and the plant in the same genus.

## Materials and Methods

### Plant Materials

The plant of *A. flaccida* was collected from Tonggubao Village, Machi Town, Changyang County, Hubei Province of China in May of 2014, and the sample was authenticated by Prof. Xuebo Hu, Assoc. Prof. Tewu Yang (College of Plant Science and Technology, Huazhong Agricultural University, Wuhan, China). All samples of *A. flaccida* were taken from a wild bush (**Figure 1A**). They are about 3 years old. The leaves and rhizomes of *A. flaccida* were rapidly separated and frozen in liquid nitrogen after collection until RNA extraction. Leaves and rhizomes from two plants were separated and each was used as a separate sample for RNA-Seq and Proteomic sequencing, thus the expression level for RNA-Seq and proteome was an average from two replicates. The rest plants were relocated to a farming field (**Figure 1B**) and some of the rhizomes were collected for germination study (**Figure 1C**). The rhizome, which is usually used as medicinal material of *A. flaccida* was shown in **Figure 1D**.

### Total RNA Extraction

Frozen tissues were transferred to a mortar pre-cooled by liquid nitrogen and grounded with pestle. Total RNA was extracted from leaves and rhizomes by Trizol Reagent (Invitrogen, USA), following the manufacturer’s instructions. The quality and quantity of RNA was checked by agarose gel electrophoresis and spectrophotometry (NanoDrop Technologies, Wilmington, DE, USA). RNA samples with A260/A280 of 1.8–2.2 were selected for cDNA synthesis and they were stored at -80°C prior to Illumina sequencing and real-time quantitative polymerase chain reaction (qRT-PCR) analysis.

### Illumina Sequencing and *De novo* Assembly Analysis of RNA-Seq

Illumina Sequencing was performed at Wuhan Bio-Broad Co. Ltd. (Wuhan, China^1^). The raw reads were cleaned by removing adaptor sequences, short reads (less than 20 nt) and reads with low-quality bases with minimum PHRED quality score threshold of 20 by ConDeTri (Smeds and Kunstner, 2011). The sequencing data of clean reads then were assembled by Trinity program (Grabherr et al., 2011). Contigs were obtained by combining reads with certain length of overlap. Then, Trinity software (default set) was used to construct unigenes with the paired end information. Unigenes sequences were aligned by Blastx (*E*-value < 1*e*^-5^) to various protein databases including non-redundant (Nr) protein database, non-redundant nucleotide (Nt), the cluster of orthologous groups (COG) database, Swiss-Prot protein database, Search Tool for the Retrieval of Interacting Genes (STRING), the Kyoto encyclopedia of genes and genomes (KEGG) pathway database. If the results of different databases were conflicted, the priority order of alignments was Nr, Nt, KEGG, Swiss-Prot databases. The unigenes were annotated according to the known sequences with the highest sequence similarity. The unigene direction was identified by the best alignment results.

Fragments per kb per million reads (FPKM) were used to show the gene expression quantity, thus avoiding the influence of sequencing length and differences. The gene expression level was calculated by the numbers of fragment mapped to the reference sequence and every gene using FPKM method (Mortazavi et al., 2008). To obtain the differential expression genes (DEGs) in the two samples, Audic and Claverie method (Audic and Claverie, 1997) were used. The false discovery rate (FDR), a useful parameter for estimation of the expected percent of false predictions in a set of predictions (Benjamini and Hochberg, 1995), was used as the threshold of *P*-value in multiple tests. For DEG significance analysis, a threshold of FDR < 0.05, | Log_2_FC|≥ 1 was used.

### The Identification of Triterpenoid Saponins Biosynthetic Genes

Candidate genes belonging to the triterpenoid saponins biosynthetic pathway in *A. flaccida* were manually identified according to annotated sequences in the above databases. Protein coding sequences (CDS) were acquired by Trinity software, and multiple sequence alignment was carried out by MEGA6.

### cDNA Synthesis and Quantitative Real-Time PCR

The first-strand cDNA was synthesized from 2 μg high-quality total RNA using a TakaRa FastQuant RT Kits (TakaRa, Japan) according to the manufacturer’s instructions, and a fivefold dilution of the resulting cDNA was used as the template for qRT-PCR analysis. Primer sequences (designed by Beacon Designer 7) were listed in **Supplementary Table S1**. QRT-PCR was performed on a Real-Time PCR instrument (Analytik Jena AG, Germany). The reaction mixture (20 μL total volume) contained 10 μL of SYBR Premix Ex Taq^TM^ (TIANGEN, China), 0.6 μL of each primer (10 μM), 2 μL of diluted cDNA and 6.8 μL of PCR-grade water. The three steps of qRT-PCR program began with 95°C for 15 min, 45 cycles of 95°C for 10 s and 59°C for 15 s, followed by 72 C 20 s and completed with a melting curve analysis with a temperature ramp from 65 to 95°C. All qRT-PCRs were repeated in three technical replicates for each sample. The qPCR results were calculated as mean of three replications. Expression levels were normalized to an internal reference gene, *elongation factor 1-alpha* (EF1α; Van Hiel et al., 2009; Saito and Ito, 2013). The relative gene expression level was calculated using the 2^-ΔΔ^*^C^*^t^ method (Livak and Schmittgen, 2001).

### Protein Extraction and Digestion

Leaves and rhizomes of *A. flaccida* were grounded with liquid nitrogen using mortars and pestles. Approximately 4 g leaves and 8 g rhizome tissue were grounded. The powdered tissue was transferred to a 50 mL ice-cold centrifuge tube. The 10% (v/v) trichloroacetic acid in acetone with 65 mM dithiothreitol was used to resuspend the fresh powder (10 ml solution per gram tissue). After complete mixing, the fresh powder was stored at -20°C for 2 h, and then the samples were centrifuged at 5,000 × *g* for 45 min at 4°C. The pellet was washed three times with 20 mL of ice-cold acetone. Protein pellet was lyophilized by vacuum. About 0.8 g of dry powder of protein was dissolved in 1.5 mL SDT lysis buffer (4% SDS, 100 mM Tris-HCl, 100 mM, dithiothreitol) in a new 1.5 mL microtube. After mixing, these microtubes were incubated at 100°C for 5 min, and then sonicated on ice and incubated at 100°C for 5 min again, followed by centrifugation at 14,000 × *g* for 45 min. Finally, the supernatant was filtrated by a 0.22 μM ultrafiltration membrane at the room temperature and then stored at -80°C for future use.

### Protein Processing and iTRAQ Labeling

Two hundred microliters of UA buffer (8 M urea, 150 mM Tris-HCl, pH 8.0) was added to each protein sample, and then the mixture was transferred to a 10-kDa ultrafiltration centrifugal tube and centrifuged at 14,000 × *g* for 30 min. The flow-through was discarded after the centrifuge. To the supernatant solution, 100 μl iodoacetamide (IAA; from BIO-RAD, 163–2109) buffer (50 mM IAA in UA buffer) were added. The mixture were vortexed and centrifuged at 4,000 × *g* for 1 min, and then incubated in the dark for 30 min at room temperature, followed by another centrifuge at 14,000 × *g* for 20 min at 4°C. The flow-through was discarded. Afterward, 100 μL UA buffer were added to the supernatant. Then the tube was span at 14000 × *g* for 20 min at 4°C and repeated twice. The flow-through was discarded after centrifuge.

For iTRAQ labeling, 100 μL dissolution buffer (4plex Application Kit, AB SCIEX) was added to the above ultrafiltration centrifugal tube and centrifugated 14,000 × *g* for 20 min at 4°C. Then 40 μL trypsin buffer (2 μg trypsin in 40 μl dissolution buffer) was added to the tube. The tube was vortexed at 4,000 × *g* for 1 min and incubated at 37°C for 16 h. A new tube was used to collect the filtrate and then centrifuge at 14,000 × *g* for 10 min at 4°C. The peptide content was examined by ultraviolet light spectral density at 280 nm (Unico WFZ UV-2100). According to the manufacturer’s instructions, the samples were labeled by the iTRAQ reagent-4plex Multiplex kit (AB SCIX). The rhizomes were labeled as iTRAQ tags 119 and the leaves were labeled as iTRAQ tags 121.

### Liquid Chromatography and Mass Spectrometry

Liquid Chromatography–Mass Spectrometry/Mass Spectrometry was performed for the SCX fractions analysis. The samples were loaded on a C18 pre-column (2 cm × 100 μm; 5 μm). Reversed phase chromatography was performed using a Thermo EASY-nLC 1000 (Thermo Fisher Scientific, Carlsbad, CA, USA) with a binary buffer system consisting of 0.1% formic acid (buffer A) and 84% acetonitrile in 0.1% formic acid (buffer B). An analytical C18 column (75 μm × 100 mm; 3 μm) was used. The online LC separation used a gradient from 0 to 35% buffer B for 200 min, then 35 to 100% buffer B for 16 min, and then 100% buffer B for 24 min. The flow rate was 250 nl/min. Peptides eluted from LC were directly injected into the coupled Q-Exactive mass spectrometer (Thermo Finnigan) via nanoelectrospray source (Thermo Finnigan). A full MS scan was operated in the data-dependent mode and acquired over the range of 300–1800 *m/z* with a mass resolution of 70000 at *m/z* 200. Up to the top ten most abundant isotope patterns with a charge of 1.6 Th, and fragmented by higher energy collisional dissociation with normalized collision energies of 27 eV. The underfilled ratio was 0.1% and the maximum ion injection times for the survey scan were 60 ms and microscans were 1. In this mode of operation, MS/MS scans were acquired with a mass resolution of 17500 at *m/z* 200 with an isolation window of 2 *m/z*. All scans were “time out” at 60 ms due to the high target value. The MS/MS data was analyzed by using Maxquant1.4.0.2 (Cox and Mann, 2008), the protein database we used were translated from the transcriptome data of *A. flaccida*. Parameter is in the default value of the software.

### Data Deposition

The raw Illumina sequencing data of *A. flaccida* was submitted to NCBI Sequence Read Archive (SRA) database, and the accession numbers for the stem and leave are SRR3233423, SRR3233424, irrespectively. This Transcriptome Shotgun Assembly project has been deposited at DDBJ/ENA/GenBank under the accession GEKW00000000. The version described in this paper is the first version, GEKW01000000.

## Results and Discussion

### Illumina Sequencing and *De Novo* Assembly

**FIGURE 3:**
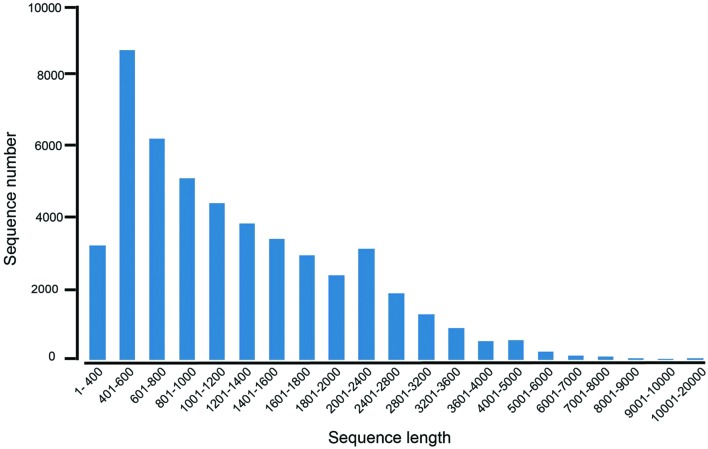
**The length distribution of unigenes from the RNA-Seq of *Anemone flaccida*.** A histogram represented the sequence-length with corresponding unigenes numbers from *A. flaccida*. The *x* axis indicates sequence sizes from 1 to 20000 nt. The *y*-axis indicates the corresponding number of unigenes.

To obtain the transcriptome overview of leaves and rhizomes in *A. flaccida*, cDNA library was constructed from an equal mixture of RNA isolated from leaves and rhizomes with pair-end sequenced of the Illumina sequencing platform. After cleaning and quality check, a total of 61,537,351 of residues were obtained and they were assembled into 36,617 genes. The average length of isogenes is 1,310 nt (**Table 1**). The lengths of assembled unigenes ranged from 351 to 15,546 nt with a N50 length of 1,704 nt (**Table 1**). The sequence length distribution of these unigenes was shown in **Figure 3**. It can be seen that more than three forth (75.94%; 35,662) of the unigenes were ranged from 601 to 2000 nt in length, indicating a comparably intact RNA-Seq manipulation and successful assembly (**Figure 3**). Of the 36,617 genes each predicted with a coding sequence, 19,439 of them have full-length open reading frames (ORFs). The complete lists of the genes, the predicted proteins and proteins in full-length were separated (**Supplementary Tables S2**–**S4**).

**Table 1:** A summary of cDNA sequencing results of *Anemone flaccida*.

Types	Numbers
Total genes	25,675
Total isogenes	46,962
Total residues	61,537,351
Average length (nt)	1,310
Largest isogene (nt)	15,546
Smallest isogene (nt)	351
N50 (nt)	1,704


### Functional Annotation of All Non-redundant

**FIGURE 4:**
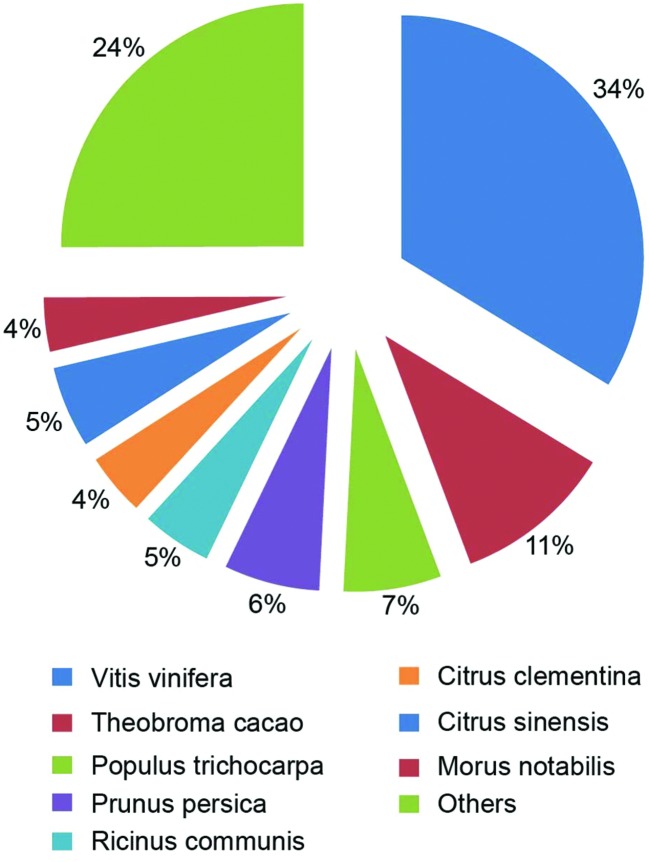
**The species distribution of the Nr annotation from the RNA-Seq of *Anemone flaccida*.** A Pie chart showed the homology of unigenes from *Anemone flaccida* with that from other species.

**FIGURE 5:**
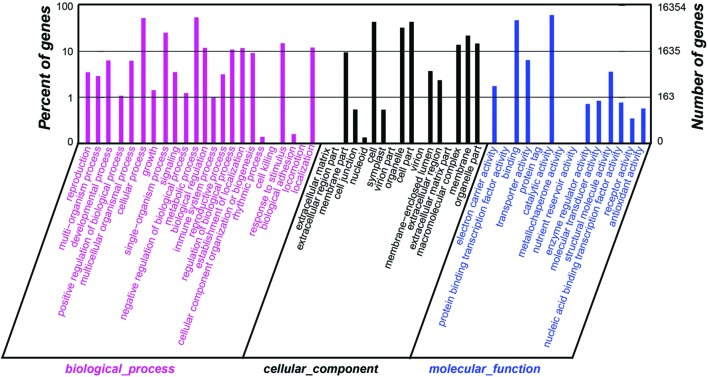
**Distributions of GO annotation of all unigenes from the RNA-Seq of *Anemone flaccida*.** The results were classified into three main categories: biological process, cellular component, and molecular function. The left *y*-axis indicated the percentage of a specific category of genes in that category. The right *y*-axis indicated the number of genes in a category.

The species distribution of the Nr annotation was shown in **Figure 4**. It showed that 34% of the unigenes had the highest homology to genes from common grape (*Vitis vinifera*), 11% to Cocoa (*Theobroma cacao*), 6% to black cottonwood (*Populus trichocarpa*), and 6% to peach (*Prunus persica*). The total sequences were annotated with COG/KOG/NOGs database and the analysis was shown in **Supplementary Table S5**. Based on Nr annotations, 16,354 unigenes were assigned to GO (gene ontology) function enrichment analysis^2^ (**Figure 5**). GO assignments were used to classify the functions of the predicted *A*. *flaccida* genes. The unigenes can be categorized into 54 functional groups in the terms of sequence homology (**Figure 5**). The GO terms are grouped into three categories (biological process, cellular component, molecular function). In the categories of biological process, ‘metabolic process’ (54.57%) is the largest group, followed by ‘cellular process’ (53.03%) and ‘single-organism process’ (25.46%). On the other hand, only a few genes form groups of ‘cell killing’ (0.036%), ‘locomotion’ (0.04%), or ‘biological adhesion’ process (0.15%; **Figure 5**). GO analysis predicted that the functions of Nr unigenes were involved in various biological processes. A total of 8,924 sequences were annotated as ‘metabolic process’ category, which suggested that the plant has a complicated secondary pathway with rich metabolites.

**FIGURE 6:**
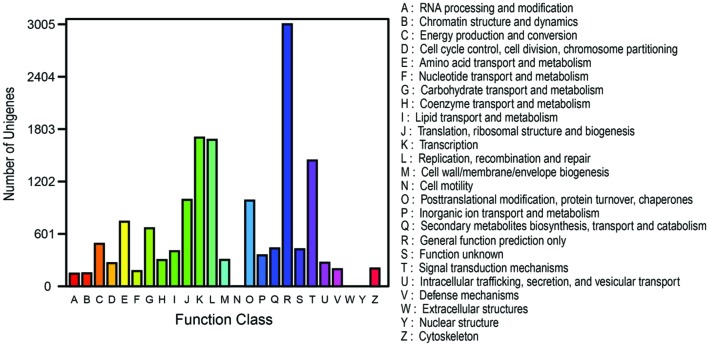
**COG function classification of all unigenes from the RNA-Seq of *Anemone flaccida*.** The annotated unigenes were divided into a variety of functional orthologous groups, of which were listed from A to Z in the right part of the figure.

To further validate the annotation effectiveness and predict possible functions of unigenes, the genes involved in the classification of Orthologous Group (COG) were examined. Out of 36,617 hits, 10,189 sequences were annotated with COG classification (**Figure 6**). The largest group of the clusters was ‘General function prediction only’ (3005 unigenes; 29.49%), followed by ‘transcription’ (1705 unigenes; 16.73%), ‘replication, recombination and repair’ (1680 unigenes; 16.49%), ‘signal transduction mechanisms’ (1443 unigenes; 14.16%), ‘translation, ribosomal structure and biogenesis’ (991 unigenes; 9.73%), ‘posttranslational modification, protein turnover, chaperones’ (983 unigenes; 9.65%), and ‘amino acid transport and metabolism’ (741 unigenes; 7.27%). The categories ‘extracellular structures’ and ‘nuclear structure’ represent the smallest groups (**Figure 6**; **Supplementary Table S5**).

To identify the active biological pathways in *A*. *flaccida*, we searched the annotated sequences in the Kyoto Encyclopedia of Genes and Genomes (KEGG) database. KEEG was employed as a reference database of pathway networks for integration and interpretation of large scale datasets generated by high-throughput sequencing technology (Wang et al., 2010; Xie et al., 2012). A total of 10,071 unigenes were assigned to 326 KEGG pathways (**Supplementary Table S6**). Among them, the ‘metabolic pathways’ (2456; 24.39% of unigenes annotated to KEGG database) was dominant, followed by ‘biosynthesis of secondary metabolites’ (1230; 12.21%). The pathways with the least representation by unique sequences were ‘vitamin digestion and absorption’ (1; 0.01%), ‘insulin secretion’ (1; 0.01%), ‘cocaine addiction’ (1; 0.01%), and ‘polyketide sugar unit biosynthesis’ (1; 0.01%; **Supplementary Table S6**).

### Putative Structural Genes Involved in the Triterpenoid Saponins Biosynthetic Pathway

High-throughput sequencing of *A. flaccida* revealed that many genes were highly enriched in the biosynthesis of secondary metabolites pathway. In the Nr annotation, 11 Nr unigenes were identified to key molecules of MVA pathway, including four for acetyl CoA *C*-acetyltransferase (AACT), one for 3-hydroxy-3-methylglutaryl CoA synthase (HMGS), three for 3-hydroxy-3-methylglutaryl CoA reductase (HMGR), one for mevalonate kinase (MK), one for phosphomevalonate kinase (PMK), one for mevalonate-5-pyrophosphate decarboxylase (MDC; **Figure 2**).

Meanwhile, nine Nr unigenes were identified as the putative genes in the MEP pathway (**Supplementary Table S7**). These genes included three unigenes for 1-deoxy-D-xylulose-5-phosphate synthase (DXPS), one for 1-deoxy-D-xylulose-5-phosphate reductoisomerase (DXR), one for 2-*C*-methyl-D-erythritol 4-phosphate cytidylyl transferase (MCT), one for 4-(cytidine 5’-diphospho)-2-*C*-methyl-D-erythritol kinase (CMK), one for 2-*C*-methyl-D-erythritol-2, 4-cyclodiphosphate synthase (MECPS), one for 4-hydroxy-3-methyl but-2-(*E*)-enyl diphosphate (HDS), and one for 4-hydroxy-3-methyl but-2-(*E*)-enyl diphosphate reductase (HDR). At the transcriptional level, most of candidate genes from the MEP pathway were up-regulated in leaves except DXPS (**Supplementary Table S7**). Three unigenes were annotated as DXPS and one of them (*comp30785_c0*) was up-regulated in rhizomes (**Supplementary Table S7**). Additionally, 46 genes were also predicted as the potential candidates for the lower part of MEP pathway, including two genes for isopentenyl diphosphate isomerase (IPPI), 13 for geranyl diphosphate synthase (GPPS), one for farnesyl diphosphate synthase (FPPS), one for squalene synthase (SS), one for squalene epoxidase (SE), six for β-amyrin synthase (β-AS), 22 for cytochrome P_450_-dependent monooxygenases (PDMO; **Supplementary Table S6**). β-AS catalyzes the cyclization of 2,3-oxidosqualene into β-amyrin, which is the precursor of oleanane-type triterpenoid saponins. In our analysis, no transcript for lupeol synthase, dammarenediol synthase, lanosterol synthase, and cycloartenol synthase was detected by RNA-sequencing (**Supplementary Table S7**). This result is consistent with the fact that oleanane-type triterpenoid saponins is the most abundant saponins in *A. flaccida*, and it has been reported that 21 triterpenoid saponins have been identified from *A. flaccida* and all of them are oleanane-type saponins (Zhao et al., 1990; Huang et al., 2014). CYP716A12 is one of the key enzyme in the oleanane-type triterpenoid saponins biosynthetic pathway involved in the oxidative reactions of the β-amyrin skeleton, which leads to oleanane-type triterpene aglycones (Carelli et al., 2011). By the RNA expression pattern and the gene annotation, we identified four candidates of CYP716A12 (*comp34900_c0*, *comp38278_c1*, *comp38278_c3*, *comp44343_c0*; **Supplementary Table S7**).

### Putative Genes Encoding Tailoring Enzymes in the Triterpenoid Saponin Downstream Pathway

It is estimated that there are more than 1,000 triterpenoid saponins (Hostettmann and Marston, 2005; Vincken et al., 2007). The structural diversity of these compounds is derived from various tailoring processes in the downstream pathway (**Figure 2**). For example, triterpenoid saponins often exist as *O*-glycosides, which are catalyzed by glycosyltransferases (UGTs). UGTs belong to one of the biggest multigene family. By keyword searching, a total of 106 unigenes were predicted to encode UGTs (**Supplementary Table S7**). Using twofold change as the threshold, 32 putative UDP-glycosyltransferases were selected as the potential candidates of triterpenoid modifiers and all of them are highly expressed in rhizomes compared to those in leaves (**Supplementary Table S7**). The multiple sequences alignment of deduced amino acids with other published plant UGTs showed that most of them share a common domain in the C-terminal region, namely, the potential UDP-binding domain (PSPG motif; **Supplementary Figure S1**). The PSPG domain is a glycosyltransferase consensus sequence of plant secondary product (Vogt and Jones, 2000). For better understanding of the UGTs phylogenetic relationship between *Arabidopsis thaliana* and other plants, we made a phylogenetic analysis. The phylogenetic analysis included a whole set of *A. flaccida* 107 UGTs, UGT71G1, UGT73K1 and UTG73F3 from *Medicago truncatula* (Achnine et al., 2005; Naoumkina et al., 2010), UGT71A27 from ginseng (*Panax ginseng*; Panax, 2014), UGT74M1 from saponaria (*Saponaria vaccaria*; Meesapyodsuk et al., 2007), UGT73C10-C13 from bittercress (*Barbarea vulgaris* subsp. *Arcuate*; Augustin et al., 2012), GT73F2 and UGT73F4 from soybean (*Glycine max* U; Sayama et al., 2012). It showed that all the above genes were clustered into 11 groups (**Supplementary Figure S2**). The function annotation indicated that most of the candidates are *O*-glycosyltransferase. These candidates are all expressed at a higher level in the stems compared to leaves, which are likely involved in the tailoring processes of the downstream pathway in the triterpenoid saponin biosynthesis.

### Validation of Illumina Sequencing by qRT-PCR

**FIGURE 7:**
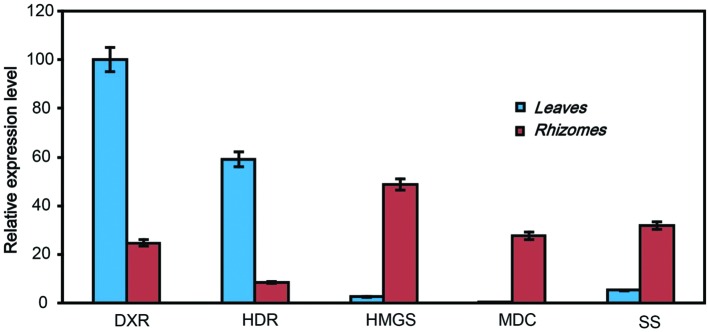
**qRT-PCR validation of the expressed genes in triterpenoid saponins biosynthetic pathway in *Anemone flaccida*.** The blue and red bars indicated the genes expression from leaves or rhizomes, irrespectively. The *y*-axis indicated the expression levels of genes relative to that of the housekeeping gene *EF1*α. The result was the mean from three replicates, and error bars indicated the standard deviation.

The qRT-PCR was used to confirm the expression profiles of genes which were identified in Illumina sequencing analysis. We selected five key genes to confirm the RNA-sequencing results by qRT-PCR. The primers set for qRT-PCR was shown in **Supplementary Table S1**. The qRT-PCR results displayed different expression abundances of these five genes in leaves and rhizomes of *A. flaccida* (**Figure 7**). Among these, the transcription of DXR and HDR genes displayed significantly higher level in leaves than that of rhizomes. On the contrary, HMGS, MDC, and SS genes showed a relative higher activity in the rhizomes of *A. flaccida*. These results were consistent with the analysis of Illumina sequencing data (**Supplementary Table S1**), and that also confirmed the reliability and accuracy of our transcriptome analysis.

### Proteome Analysis of *A. flaccida*

**FIGURE 8:**
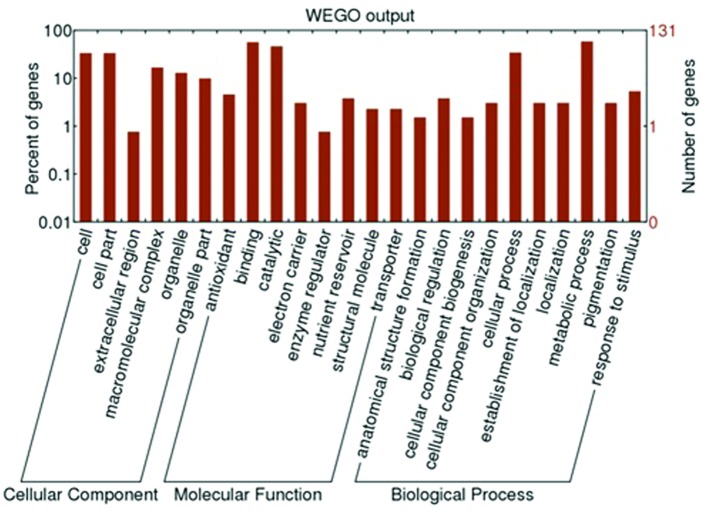
**GO enrichment analysis for the genes of interest in *Anemone flaccida*.** The enriched GO “biological process,” “cellular components,” and “molecular function” categories were shown. Further classification under each category was also listed.

The LC-MS/MS data analysis revealed that 4902 unique peptides, which were assembled into 1517 proteins, were identified (**Supplementary Table S8**). According to transcriptome and proteome data analysis, 1473 genes were identified from both mRNA sequences and proteins. To confirm the differentially expressed proteins between leaves and rhizomes of *A. flaccida*, proteins with 1.5-fold changes were considered as differentially expressed proteins. In this regard, 129 proteins (8.6%) demonstrated statistically significant (*P* < 0.05) differential expression between leaves and rhizomes of *A. flaccida* (**Supplementary Table S9**). All the identified proteins and the 129 differential expressed proteins were functional annotated by GO and KEGG database (**Supplementary Table S10**). The 129 proteins were divided into 24 functional groups, which were divided into three categories: cellular component, molecular function and biological process (**Figure 8**). The proteins involved in cell and cell part, binding and catalytic, cellular process and metabolic process took higher percentages, which was consistent with the transcriptomes analysis (**Figure 8**).

For further exploration of the triterpenoid saponins biosynthetic pathway of *A. flaccida*, unigenes involved in the pathway were selected through KEEG annotation. In the translation level, 14 unigenes were identified from the expressed protein table (**Supplementary Table S11**). These unigenes were matched to the function genes which involved in the metabolic pathway of triterpenoid saponins biosynthesis. Twelve unigenes (*comp39291_c0*, *comp38705_c0*, *comp29437_c0*, *comp31609_c0*, *comp39787_c0*, *comp26165_c0*, *comp36650_c0*, *comp29613_c0*, *comp29171_c0*, *comp29722_c0*, *comp24447_c0*, and *comp21171_c0*) showed highest identity with HDS, DXR, AACT, HMGS, DXPS, HDR, HMGR, MDC, CMK, MECPS, IPPI, and MCT genes, respectively. Meanwhile, *comp24200_c0* and *comp16859_c0* were corresponded to the GPPS gene (**Supplementary Table S10**). Of the 14 proteins were found in different quantities in the triterpenoid saponins biosynthetic pathway, 11 of them were upregulated in rhizomes (**Supplementary Table S11**).

### Comparative Analysis of Proteome and Transcriptome Data

**FIGURE 9:**
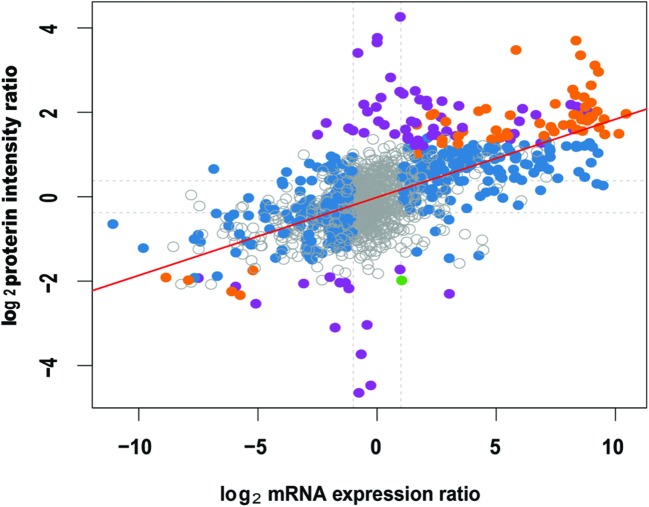
**Correlation analysis of the overall proteome and transcriptome of *Anemone flaccida*.** The differentially expressed mRNA or protein was represented by solid dots (*n* = 1473). The red line indicates a linear correlation between the protein and mRNA (*r* = 0.619, *p*-value < 2.2*e* - 16).

For the comparative analysis of proteome and transcriptome data, we focus on the 14 unigenes that are both detected in transcriptome and proteome sequencing (**Supplementary Table S11**). Different trend was observed between protein and transcription levels. For instance, *comp39291_c0*, *comp29437_c0*, *comp39787_c0*, *comp29171_c0*, *comp29722_c0*, *comp24447_c0*, *comp21171_c0*, and *comp16859_c0* were downregulated at the transcriptional level but upregulated at the protein level (**Supplementary Table S11**). On the other hand, the fold change between the protein and transcription level was not proportional, which was indicated by the low correlation coefficient of the overall proteome and transcriptome data (*r* = 0.619 *p*-value < 2.2*e* - 16; **Figure 9**). It might be because the mRNA quantity is not always consistent with protein levels due to the posttranscriptional, translational and/or posttranslational regulations (Washburn et al., 2003).

## Conclusion

In the present study, a tandem transcriptome and proteome profiling approach was presented to show the difference between leaves and rhizomes of *A. flaccida*. Our research provided a comprehensive overview of the proteins/genes in the pathway of triterpenoid saponins synthesis in *A. flaccida*. As far as we know, this is the first publication about the transcriptome and proteome profiling of *A*. *flaccida* by Illumina sequencing and iTRAQ technology. This study would be helpful to understand the mechanism of triterpenoid saponins biosynthesis in *A. flaccida* at the molecular level. It is also the first time to discover the genetic and protein information of the genus of *Anemone*, which provides valuable clues for understanding of the plants in the same group. Our study shall greatly help further molecular cloning and functional identification of triterpenoids biosynthesis genes in *A. flaccida.* Based on assumption, P450s and UGTs with unknown functions are highlighted in our analysis because they are most likely the committed enzymes for those unclear steps in the triterpenoid biosynthesis pathway. Given a very low growth and biomass accumulation of the plant, it is expected that triterpenoid pathway may be engineered in model species such as yeast to product rare triterpenoid saponins from *A. flaccida* in a large-scale.

## Author Contributions

Conceived and designed the experiments: XH, XL, and CZ. Performed the experiments: CZ, BL, QZ, and YH. Analyzed the data: CZ and XH. Performed sample preparation and experiments: ZZ, XW, CZ, and TY. Wrote the paper: CZ, XL, and XH.

## Conflict of Interest Statement

The authors declare that the research was conducted in the absence of any commercial or financial relationships that could be construed as a potential conflict of interest.

## Footnotes

1 http://www.bio-broad.com/

2 http://www.geneontology.org/

## Supplementary material

FIGURE S1 **Multiple alignment of deduced amino acid sequences of UGTs from *Anemone flaccida* and other species (representative kinds only).** Black shaded and gray shaded boxes show identical and similar amino acids, respectively. The conserved PSPG motif at the C-termini is indicated by the green box.

FIGURE S2 **Phylogenetic analysis of UGTs from* Anemone flaccida* and other species (representative kinds only).** The UGTs from *Anemone flaccida* was marked as green solid dots.

TABLE S1 **Primers used for qRT-PCR in the study**.

TABLE S2 **A complete list of the coded genes in *Anemone flaccida***.

TABLE S3 **A complete list of the predicted proteins in *Anemone flaccida***.

TABLE S4 **A complete list of the predicted proteins in full-length in *Anemone flaccida***.

TABLE S5 **Sequences of *Anemone flaccida* by COG/KOG/NOG analysis**.

TABLE S6 **Unigenes of *Anemone flaccida* annotated by KEEG**.

TABLE S7 **Differential expressed genes involved in triterpenoid between leaves and rhizomes by RNA-Seq**.

TABLE S8 **The identification and quantification result of proteins from *Anemone flaccida*.** A total of 1517 was identified, including 129 differentially expressed proteins in the leaves versus the rhizomes (yellow color).

TABLE S9 **Differentially expressed proteins from leaves and rhizomes of *Anemone flaccida*.** A total of 127 genes were expressed differentially at the translation level.

TABLE S10 **All identified proteins and the 129 differential expressed proteins functional annotated by GO and KEGG database**.

TABLE S11 **The putative genes detected at both transcriptional and protein level**.
