# Supplementary material for: Comprehensive Analysis of the Triterpenoid Saponins Biosynthetic Pathway in Anemone flaccida by Transcriptome and Proteome Profiling
Source: Front Plant Sci. 2016 Jul 25;7:1094. doi: 10.3389/fpls.2016.01094 (PMC4958654; doi:10.3389/fpls.2016.01094)
Supplement: FIGURE S1 — Multiple alignment of deduced amino acid sequences of UGTs from Anemone flaccida and other species (representative kinds only). Black shaded and gray shaded boxes show identical and similar amino acids, respectively. The conserved PSPG motif at the C-termini is indicated by the green box. [file Data_Sheet_1.ZIP › supplement/Figure Supplementary 1.rtf]

comp26945_c0    1 QMDSFVGNESWRIRP-----------------Y-----ARKGDNTAMGSV----------
comp38054_c0    1 ------------------------------------------------------------
comp36880_c0    1 ------------------------------------------------------------
comp38259_c0    1 ------------------------------------------------------------
comp38889_c0    1 ------------------------------------------------------------
comp36259_c0    1 ------------------------------------------------------------
comp38694_c0    1 --------HVWHLAVVIIEITEIHLHPCVVLTVSEW---S-GELISMAGRRGT----VSA
comp23425_c0    1 -----------------------------------------------PGKKSVFKKLIVM
comp37829_c0    1 ----------------------------------------------------------ME
PgUGT71A27      1 ------------------------------------------------------------
MtUGT71G1       1 ---------------------------------------------------------MSM
SvUGT74M1       1 ----------------------------------------------------------MS
comp37573_c0    1 ------------------------------------------------------------
comp31343_c0    1 ---------------------------------------------------REQKMEDAN
comp36943_c0    1 ------------------------------------------------------------
BvUGT73C10      1 -------------------------------------------------------MVSEI
BvUGT73C11      1 -------------------------------------------------------MVSEI
BvUGT73C12      1 -------------------------------------------------------MVSEI
BvUGT73C13      1 -------------------------------------------------------MVSEI
MtUGT73K1       1 -----------------------------------------------------------M
MtUTG73F3       1 ---------------------------------------------------------MEG
GmUGT73F4       1 -----------------------------------------------------------M
GmUGT73F2       1 -----------------------------------------------------------M
comp26042_c0    1 ------------------------------------------------------------
comp35666_c0    1 ------------------------------------------------------------
comp35395_c0    1 ------------------------------------------------------------
comp32485_c0    1 ----------------------------------------------MDKRRGKKAIQHNN
comp27554_c0    1 ---------------------------------------------------------MSK
comp32214_c0    1 ------------------------------------------------------------
comp34116_c0    1 ------------------------------------------------------------
comp34883_c0    1 ------------------------------------------------------------
comp32596_c1    1 --------------------------------------------MLIGMEYVLLFKRLYK
comp38012_c0    1 ----------------------------------------------MGCAPL---FQNVK
comp34587_c0    1 ------------------------------------------------------------
comp21103_c0    1 --------------LSSFNNGDAHKPNRLLLQWGSLGNASFVIFRSMGV---DSHSRMCQ
comp22969_c0    1 ------------------------------------------------------------
comp31694_c0    1 --------------------------------------------MAMGFHSPVDIQKMCH
comp32596_c0    1 ------------------------------------------------------------
comp30460_c1    1 ------------------------------------------------------------
comp30728_c0    1 ----------------------------------------------MGRRAADFRRAGGR
comp35926_c0    1 -----------------------HFKSQILYLFTPK--YQILSATAMAGGV-RGTRQGGS
comp29055_c0    1 ------------------------------------------------------------
comp32966_c0    1 ------------------------------------------------------------

consensus          1                                                             


comp26945_c0   29 TELSVKP----IV-GDQVGPRCSINHSIPAVVFS-SGGYAGNHF-------HDFADIIVP
comp38054_c0    1 ------------------------------------------------------------
comp36880_c0    1 -MSSFPL-------HVAMLPWFATGHITPYIHLSNKFAEQGHR-VSFF--LPTKTQTKFI
comp38259_c0    1 --MKSSL-------HIVMFPWLAFGHMLPFFQLSKHLASRGNR-VSFV--STPRNISRLP
comp38889_c0    1 -MAGEDL-------HIVMFPWLAFGHLNPFFQLAKCLANKGHR-ISFV--STPRNISRLP
comp36259_c0    1 ---MGRP-------HLLVVPFPAQGHVMPLMELSHNLVERGFK-ITFV--NSEFNHKRVM
comp38694_c0   45 TGEQQRR-------RVALFPCPFQGHITPMLQLATVLHSHSKGALSII--IVHPQFNA--
comp23425_c0   14 EEEKPVP-------HVLIVPLPFQSHVNNMLRLAELLCMSDLH-VTFL--ITHQNYHSLH
comp37829_c0    3 EQKPAQP-------HVLMVPFLLPGHINPMLQLAELLCLSGLQHVTFV--CTKYHHNRLL
PgUGT71A27      1 ----MKS-------ELIFLPAPAIGHLVGMVEMAKLFISRHEN-LSVTVLIAKFYMDTGV
MtUGT71G1       4 SDINKNS-------ELIFIPAPGIGHLASALEFAKLLTNHDKN-LYITVFCIKFPGMPFA
SvUGT74M1       3 NNENNAT-------QVIVLPYHGQGHMNTMVQFAKRLAWKGVH-VTIA--TTFNTIQQMK
comp37573_c0    1 --------------------------------------------ITFA--TTVYFSKSMH
comp31343_c0   10 NQIRNRV-------HVLVLPYPIQGHINPMLQFAKRLVSKGID-VTLA--ITAFTAKSTP
comp36943_c0    1 -MENNNP-------HVLVLPFHGQGHINPMLQFSKRLASKGVK-ITLV--STLSSIKTMP
BvUGT73C10      6 THKSYPL-------HFVLFPFMAQGHMIPMVDIARLLAQRGVK-ITIV--TTPHNAARFE
BvUGT73C11      6 THKSYPL-------HFVLFPFMAQGHMIPMVDIARLLAQRGVK-ITIV--TTPHNAARFE
BvUGT73C12      6 THKSYPL-------HFVLFPFMAQGHMIPMVDIARLLAQRGVK-ITIV--TTPHNAARFK
BvUGT73C13      6 THKSYPL-------HFVLFPFMAQGHMIPMVDIARLLAQRGVK-ITIV--TTPHNAARFE
MtUGT73K1       2 GTESKPL-------KIYMLPFFAQGHLIPLVNLARLVASKNQH-VTII--TTPSNAQLFD
MtUTG73F3       4 VEVEQPL-------KVYFIPFLASGHMIPLFDIATMFASRGQQ-VTVI--TTPANAKSLT
GmUGT73F4       2 DLQQRPL-------KLHFIPYLSPGHVIPLCGIATLFASRGQH-VTVI--TTPYYAQILR
GmUGT73F2       2 DLQQRPL-------KLHFIPYLSPGHVIPLCGIATLFASRGQH-VTVI--TTPYYAQILR
comp26042_c0    1 ------------------------------------------------------------
comp35666_c0    1 ------------------------------------------------------------
comp35395_c0    1 ------------------------------------------------------------
comp32485_c0   15 TDDN-NIWWMLERGRVSWILCI--------TVFALL------------------IRVIVS
comp27554_c0    4 VAME-DLVVLVKIG--IILSFI--------IASASASAS------------AQVDSKLHI
comp32214_c0    1 --------MSLRFG--LLFLSI-------------AFLS------------TPALSNLVI
comp34116_c0    1 ------------------------------------------------------------
comp34883_c0    1 ------------------------------------------------------------
comp32596_c1   17 VEAR-TLF--SFLGALTMCVIL--------VQT-LT---------------LPHRFGFSG
comp38012_c0   12 FDAK-RSI--LVVGVLFSVVLV--------LQS-SS---------------IPYEDIISS
comp34587_c0    1 ------------------------------------------------------------
comp21103_c0   44 LDIR-KLVFVII--PVAAIVLM--------VQF-VS---------------VPYGNVLLS
comp22969_c0    1 ------------------------------------------------------------
comp31694_c0   17 LDTR-KLVFLIILTSVAVIVFM--------LQF-LS---------------FPYGSVPLI
comp32596_c0    1 ------------------------------------------------------------
comp30460_c1    1 ------------------------------------------------------------
comp30728_c0   15 RRLSKVFWL--TLGGVCVLLFI--------VVL-SRDGS------------K--------
comp35926_c0   35 SMLRNIFSYRIFVSAMFTLLFI--------ATL-SIIFT------------TNTNHESV-
comp29055_c0    1 -MA--NTSLSIFLTSFLLLPFL--------T-I-SFTPE------------NPTNKQILV
comp32966_c0    1 -------MFPLFLRSM-----C--------K-C-YL---------------KPKQRPPLF

consensus         61                . . .          .                             


comp26945_c0   76 LFVTSR--------------------------------------------------QFDG
comp38054_c0    1 ------------------------------------------------------------
comp36880_c0   50 SLNLH----PHLFQFI--------------------------------------------
comp38259_c0   49 KIPSDL---ASLFHYVE-------------------------------------------
comp38889_c0   50 NIPLDI---ASLLTFVE-------------------------------------------
comp36259_c0   48 AALSD----KENSVKDS-------------------------------------------
comp38694_c0   94 ---------PNPSKYPD-------------------------------------------
comp23425_c0   64 HHGDTQ---TRFTSFPK-------------------------------------------
comp37829_c0   54 SFTDAQ---TRFAYFPG-------------------------------------------
PgUGT71A27     49 DNYNKSLLTNPTPRLTI-------------------------------------------
MtUGT71G1      56 DSYIKSVLA-SQPQIQL-------------------------------------------
SvUGT74M1      53 LNISSY----NSITLEP-------------------------------------------
comp37573_c0   15 LKDC-------EFDVEL-------------------------------------------
comp31343_c0   60 PQTG-------SIRINT-------------------------------------------
comp36943_c0   50 PQVD-------SISFES-------------------------------------------
BvUGT73C10     56 NVLSRAIESGLPISIVQ-------------------------------------------
BvUGT73C11     56 NVLSRAIESGLPISIVQ-------------------------------------------
BvUGT73C12     56 NVLSRAIESGLPISIVQ-------------------------------------------
BvUGT73C13     56 NVLNRAIESGLPISIVQ-------------------------------------------
MtUGT73K1      52 KTIEEEKAAGHHIRVHI-------------------------------------------
MtUTG73F3      54 KSLSSD--APSFLRLHT-------------------------------------------
GmUGT73F4      52 KS-------SPSLQLHV-------------------------------------------
GmUGT73F2      52 KS-------SPSLQLHV-------------------------------------------
comp26042_c0    1 ------------------------------------------------------------
comp35666_c0    1 ------------------------------------------------------------
comp35395_c0    1 ------------------------------------------------------------
comp32485_c0   48 MHP---------------------------------------------------------
comp27554_c0   41 VNAERR------I-----------------------------------------------
comp32214_c0   26 SKVDRR------I-----------------------------------------------
comp34116_c0    1 ------------------------------------------------------------
comp34883_c0    1 ------------------------------------------------------------
comp32596_c1   50 LFPNNK---IVIHKDVE-----------------------------------SNS-----
comp38012_c0   45 LLSFK-----PVLIGHSKFP--------------------------------NGSCCIDS
comp34587_c0    1 ------------------------------------------------------------
comp21103_c0   77 IFPPEK---FPLLVQTEDSP--------------------------------ANS-SPEA
comp22969_c0    1 ------------------------------------------------------------
comp31694_c0   52 VFPSEK---SPLLAQSEHKA--------------------------------ADS-SPEA
comp32596_c0    1 ------------------------------------------------------------
comp30460_c1    1 ------------------------------------------------------------
comp30728_c0   44 --PISR---SGSISQRTYRQNRMSDSLNVTEEMLSPDSVTRQVGDQIALAKAFVVIAKE-
comp35926_c0   73 LLPPSS---TTTLD-HNYIHRTF-LALKSNPAKTRFDLIHKQANDHIALVNVYSSYARK-
comp29055_c0   36 LLDDSSIKSSHSIFFNSLQQRGYSLDFKLAD-------------DPKISLNRYGQYLYEA
comp32966_c0   24 LLRVKSSSSSSYASFNPLTAM---------------------------------------

consensus        121                                                             


comp26945_c0   86 EVQFL-VTNVKQWWLSK-------------------------------------------
comp38054_c0    1 ------------------------------------------------------------
comp36880_c0   62 -------------PLTI-------------------------------------------
comp38259_c0   63 ------------LLLPR-------------------------------------------
comp38889_c0   64 ------------IQLPS-------------------------------------------
comp36259_c0   61 ------------ICLVT-------------------------------------------
comp38694_c0  102 ------------FTFLP-------------------------------------------
comp23425_c0   78 ------------FCFQT-------------------------------------------
comp37829_c0   68 ------------FRFET-------------------------------------------
PgUGT71A27     66 ------------VNLPE-------------------------------------------
MtUGT71G1      72 ------------IDLPE-------------------------------------------
SvUGT74M1      66 ------------IYDDT-------------------------------------------
comp37573_c0   25 ------------FSDGF-------------------------------------------
comp31343_c0   70 ------------FSDGF-------------------------------------------
comp36943_c0   60 ------------IYDDV-------------------------------------------
BvUGT73C10     73 ------------VKLPS-------------------------------------------
BvUGT73C11     73 ------------VKLPS-------------------------------------------
BvUGT73C12     73 ------------VKLPS-------------------------------------------
BvUGT73C13     73 ------------VKLPS-------------------------------------------
MtUGT73K1      69 ------------IKFPS-------------------------------------------
MtUTG73F3      69 ------------VDFPS-------------------------------------------
GmUGT73F4      62 ------------VDFPA-------------------------------------------
GmUGT73F2      62 ------------VDFPA-------------------------------------------
comp26042_c0    1 ---MAPS---FQWWV-----------------------KESHRGT----------P----
comp35666_c0    1 ---MAPSLDFSDWWA-----------------------KDVRKGT----------P----
comp35395_c0    1 ------------------------------------------------------------
comp32485_c0   51 ------------------------------------------------------------
comp27554_c0   48 ---------------------------------------DLSSH----------------
comp32214_c0   33 ---------------------------------------DLTSQ----------------
comp34116_c0    1 ------------------------------------------------------------
comp34883_c0    1 ------------------------------------------------------------
comp32596_c1   67 SVR--------------------------LEL------VEIREGSVREGQGLD---VK--
comp38012_c0   68 SVAL-----------NV---SN------DVEL------QK--------------------
comp34587_c0    1 ------------------------------------------------------------
comp21103_c0  101 EIVFAISTNSPGLELDI------------------------------EGVGNVREPV---
comp22969_c0    1 ------------------------------------------------------------
comp31694_c0   76 EVLFGISLSNASISSNSSSIGE------DVNA------ADQRKGM--EGVGDDGSEVDED
comp32596_c0    1 ------------------------------------------------------------
comp30460_c1    1 ------------------------------------------------------------
comp30728_c0   98 -------SNNLQFAW------------------------ELSAQIR--------------
comp35926_c0  127 -------L-----KL------------------------DNSKQLRV----------FED
comp29055_c0   83 LILFSPSVTKFGGSLDAASILAFVDAGHDLILAADSNPSDLIREIAV----ECGVDFDED
comp32966_c0   45 ---------------------------------AKDQSQNLSKKIHR----FCSLMVFVS

consensus        181                                                             


comp26945_c0  102 ----YQRILSSLSRYDIIDMDKE-------------------------------------
comp38054_c0    1 ----------------------------------------------------------MQ
comp36880_c0   66 ------------------KLEGLPDGA--ETMADVDLDQISKIQKAFDLTQQQ--L----
comp38259_c0   68 ------------------DPDNLPDGA--ESTNDVPLDKVEYLKKAMDGLEPS--L----
comp38889_c0   69 -------------------VQHLPEGA--EATSDLPLNKVPYLKTAYDGLEPA--M----
comp36259_c0   66 ------------------IPDGMEPGE--DRN--DIGKLIDAMAIVAAGHLEELIK----
comp38694_c0  107 ------------------VSDGLSAET--IAQLPDPAAVIRLLNESCVSSFKDC-L----
comp23425_c0   83 ------------------ITDGLPDDH--SRTMDKASEMLSALQSGTKPALRQLLI----
comp37829_c0   73 ------------------IPDGLPDEH--TRTFSQIEDIFNGIRSVFEPSFQELLF----
PgUGT71A27     71 ------------------TDPQNYML--KPRHAI-FPSVIETQK----THVRDIIS----
MtUGT71G1      77 ------------------VEPPPQELLKSPEFYI--LTFLESLI----PHVKA-------
SvUGT74M1      71 ------------------DDSTLH-IK--DRMAR-----FEAEA---ASNLTRVLEAK--
comp37573_c0   30 ------------------DETGPF-GA--ASGAV-FLSTITEVG---SRTLSDVINN---
comp31343_c0   75 ------------------DERGLA-QA--GSIDA-YREGFNTVG---RQTISDLIAT---
comp36943_c0   65 ------------------SNGGFN-GE--GGLMG-FMQRFQSAG---GEYIRDLVKR---
BvUGT73C10     78 ------------------QEAGLPEGN--ETFDS-LVSTKLLVP---FFKAVNMLEEP--
BvUGT73C11     78 ------------------QEAGLPEGN--ETFDS-LVSMELLVP---FFKAVNMLEEP--
BvUGT73C12     78 ------------------QEAGLPEGN--ETLDS-LVSMELMIH---FLKAVNMLEEP--
BvUGT73C13     78 ------------------QEAGLPEGN--ETFDS-LVSMELLVP---FFKSVNMLEEP--
MtUGT73K1      74 ------------------AQLGLPTGV--ENLFA-ASDNQTAGK---IHMAAHFVKAD--
MtUTG73F3      74 ------------------QQVGLPEGI--ESMSS-TTDPTTTWK---IHTGAMLLKEP--
GmUGT73F4      67 ------------------KDVGLPDGV--EIKSA-VTDLADTAK---FYQAAMLLRGP--
GmUGT73F2      67 ------------------KDVGLPDGV--EIKSA-VTDLADTAK---FYQAAMLLRRP--
comp26042_c0   18 ----VVVKMDN-PNYQISELQGPE------------------------------------
comp35666_c0   21 ----VVVTMEN-PNYSLLEIDSPD------------------------------------
comp35395_c0    1 -----------------------------------------------------MV-----
comp32485_c0   51 ------------------------------------------------------------
comp27554_c0   53 ------------------------------------------------------------
comp32214_c0   38 ------------------------------------------------------------
comp34116_c0    1 ------------------------------------------------------------
comp34883_c0    1 ------------------------------------------------------------
comp32596_c1   90 ------------------------------------------------------------
comp38012_c0   82 ----------------------SNDGG--IKLILG-NASSVETSSISQSLA---------
comp34587_c0    1 ------------------------------------------------------------
comp21103_c0  128 ----------------------HDLPS--ENVSFE-DVEKETNITLERILAPAL------
comp22969_c0    1 --------MKNLFMYVMKEPRRHCRG---YRMSWR-------------------------
comp31694_c0  122 FDPETEVSLEEVIQFGDLEQSQDSSGS--KNVSFD-NVQNESNLE---------------
comp32596_c0    1 ------------------------------------------------------------
comp30460_c1    1 ------------------------------------------------------------
comp30728_c0  113 ---NSQILLSN--------------AA--TRRSPLTARESETTIRD---MALLLYQAQQQ
comp35926_c0  141 LTRNFTELLTR-PSYKIFDTD----QA--IDEDVL--RQFEKEVKDKVKIARQLISDAKE
comp29055_c0  139 --A-SAVVIDH-ANYAEKDGD----------HSLIVSDDF---ILSHAILGRDMIQA---
comp32966_c0   68 LIS-SVSVLYW-SHY----SD----------SSWFSQEHF---FSV--------QPE---

consensus        241                                                             


comp26945_c0  121 -------------------------------------------TEVHCFSS---------
comp38054_c0    3 SFGNV--------------------F-MQGSRVSR------HVTETICPPLK--------
comp36880_c0  100 -----------------STLLS-----------------TLKPDFIFYDFA---------
comp38259_c0  102 -----------------AKFLE-----------------TSTPDWIVYDFMH--------
comp38889_c0  102 -----------------TNILE-----------------VSSPDWLICDFAS--------
comp36259_c0  100 -----------------KINDS-----------------SHGDDRITGVVADVLV-----
comp38694_c0  142 -----------------ANEIS-----------------KQNEPPVSCIITDAWM-----
comp23425_c0  119 -----------------SNYFE-----------------SINRPPVTCMIIDGIMA----
comp37829_c0  109 -----------------SDHFK-----------------SANRPPVTCIITDGMF-----
PgUGT71A27    102 -------------------GMT-----------------QSESTRVVGLLADLLF-----
MtUGT71G1     106 -------------------TIK-----------------TILSNKVVGLVLDFFC-----
SvUGT74M1     100 -----------------KQQQ--------------------ALNKKCLLVYHGSL-----
comp37573_c0   62 -----------------YNSNP-------------------DHEPINCLVCDSIL-----
comp31343_c0  107 -----------------QQKQS-----------------LNELKSFSCLVYDAYI-----
comp36943_c0   97 -----------------YQD---------------------SSSPVKCLVYDGNM-----
BvUGT73C10    112 -----------------VQKLF-----------------EEMSPQPSCIISDFCL-----
BvUGT73C11    112 -----------------VQKLF-----------------EEMSPQPSCIISDFCL-----
BvUGT73C12    112 -----------------VQKLF-----------------EEMSPQPSCIISDFCL-----
BvUGT73C13    112 -----------------VQKLF-----------------EEMSPQPSCIISDFCL-----
MtUGT73K1     108 -----------------IEEFM-----------------KEN--PPDVFISDIIF-----
MtUTG73F3     108 -----------------IGDFI-----------------END--PPDCIISDSTY-----
GmUGT73F4     101 -----------------IAHFM-----------------DQH--PPDCIVADTMY-----
GmUGT73F2     101 -----------------ISHFM-----------------DQH--PPDCIVADTMY-----
comp26042_c0   37 ----------------------DEDFMGNGGGNEGSS--SG-------GG----------
comp35666_c0   40 ----------------------AP------------------------FQ----------
comp35395_c0    3 ------------------QESIS--ALKRGGAAST----PALPTTTTARRA---------
comp32485_c0   51 ------------------------------------------------------------
comp27554_c0   53 -------IVKTFFTLEFQNEAEE----------------AQVESILLAFSPTQ-------
comp32214_c0   38 -------IVRIFSTLKVENEGSD----------------T-VSEVLLCFPDYQ-------
comp34116_c0    1 ------------------------------------------------------------
comp34883_c0    1 ------------------------------------------------------------
comp32596_c1   90 ----------------------DDVVLGRSGGASLVEKVPDHDNILKEM-----------
comp38012_c0  108 --------------PSPLEATVDLNSLRQSGANSSKHDVSSISTKATE------------
comp34587_c0    1 ------------------------------------------------------------
comp21103_c0  157 ------PPVGLSNILAPLDGPSDNSA------VSADANISSLGEQATNMK--------SQ
comp22969_c0   25 ------------------------TV------MSAVVVIATIGAFIQTFVIDFPLHKWIL
comp31694_c0  164 -------LVKDQSILSPLHSNLESTS------LSADANISSIGEQTKSMM--------SI
comp32596_c0    1 ------------------------------------------------------------
comp30460_c1    1 ------------------------------------------------------------
comp30728_c0  151 HYDSATMIMKLKAKIQSLEEQSSSVS--EKSSKYGQIAAEEVPKSLYCLGVRLTVEWFRN
comp35926_c0  192 SFDNQIKIQKLKDTIFAVNEQLSKTK--KHGAFSSLIAAKSMPKSLHCLAMRLVGEKIAH
comp29055_c0  179 ----PVLFKGIAHTLNPANSQILKVL--SA----SESAYSANPDTKLSSPPSLTGSKISL
comp32966_c0   98 ----PIYLPSYPSAW-------------------NHLTFSSTP------PPKLLK-----

consensus        301                                                 .           


comp26945_c0  129 ---------------------------------------------V--------------
comp38054_c0   28 ---------------------------RI-SATAGRTAAAALFVTVSSLIVIAVVYSFYI
comp36880_c0  117 --------------------YWMPSIAKP------LGIKSIHLSAVSVGSLVG----L--
comp38259_c0  120 --------------------SWVPPLATK------HGVQQMFFGIFVAACLAF----MGP
comp38889_c0  120 --------------------YWLPLVAAK------CNIPCAFFSIFNAANISF----FGT
comp36259_c0  121 --------------------GAAIEVAEK------LLIPRAAFWPASCLLLAE----LLH
comp38694_c0  163 --------------------YFTQSVADD------LQLPRLVLRTGSALNLFI----CLS
comp23425_c0  141 --------------------SIGVDIAVE------LEIPSIVYRTPSACYLWC----CYC
comp37829_c0  130 --------------------SFPIAVAMD------LGIHSIDFRVTNAAFLWS----LYR
PgUGT71A27    121 --------------------INIMDIANE------FNVPTYVYSPAGAGHLGL----AFH
MtUGT71G1     125 --------------------VSMIDVGNE------FGIPSYLFLTSNVGFLSL----MLS
SvUGT74M1     118 --------------------NWALVVAHQ------QNVAGAAFFTAASASFAC----YYY
comp37573_c0   81 --------------------PWALDVGKK------FGLITASFYTQPIAVSSI----FYH
comp31343_c0  128 --------------------PWALDLAKD------FGLVGAPFFTQSCAINNI----YYH
comp36943_c0  114 --------------------PWGLDVAKE------VGILGAAFFTQSCAAIAS----YLE
BvUGT73C10    133 --------------------PYTSKIAKK------FNIPKILFHGMCCFCLLC----MHV
BvUGT73C11    133 --------------------PYTSKIAKK------FNIPKILFHGMCCFCLLC----MHV
BvUGT73C12    133 --------------------PYTSKIAKK------FNIPKILFHGMCCFCLLC----MHI
BvUGT73C13    133 --------------------PYTSKIAKK------FNIPKILFHGMCCFCLLC----MHV
MtUGT73K1     127 --------------------TWSESTAKN------LQIPRLVFNPISIFDVCM----IQA
MtUTG73F3     127 --------------------PWVNDLADK------FQIPNITFNGLCLFAVSL----VET
GmUGT73F4     120 --------------------SWADDVANK------LRIPRLAFNSYPLFAVSA----MKS
GmUGT73F2     120 --------------------SWADDVANN------LRIPRLAFNGYPLFSGAA----MKC
comp26042_c0   56 -------GRDKGRGKNAKQLTWVLLLKAHKA----AG---CL-TSIAS-TLFG----LGT
comp35666_c0   44 -------PVDKDRGKNAKQFTWVLMLKAHRA----VG---CL-TWLAT-MLWA----LLG
comp35395_c0   30 -------------------------------SSRGRQIHKTF-NNVKITILCG----FVT
comp32485_c0   51 ------------------------------------------------------------
comp27554_c0   83 -------VDN-------I---------------------------------AL----L--
comp32214_c0   67 -------AKN-------L---------------------------------AF----L--
comp34116_c0    1 --------------------------------------MRIL-E--------------SS
comp34883_c0    1 ------------------------------------------------------------
comp32596_c1  117 ------VSRNDSV----------------------------E-Q--------------KP
comp38012_c0  142 ---THSLSDN------------------------------------------------AE
comp34587_c0    1 ------------------------------------------------------------
comp21103_c0  197 HNTTEILENNRDTLHNNVTFTL---------QNNGRGTSRIE-QN---------------
comp22969_c0   55 DPPVNIFS---GE------------------VS--NGNLLVK-ETVS-----------SA
comp31694_c0  203 DKTPEILGNNQGTSENNSSMTSISVMKDKSDVPTGRGNTRIA-FPKT-----------SN
comp32596_c0    1 ------------------------------------------------------------
comp30460_c1    1 --------------------------------------MFRL-TSALLFLFFF----FIN
comp30728_c0  209 IHIQEKLTED-------------------------SRAVEIL-TDNNRYHFCV----FSD
comp35926_c0  250 ---PEKYVDN--------------------------GYKAEF-ENPSLYHYAI----FSD
comp29055_c0  229 VSVVQAR--N-------------------------NA--RIL-ISGS---LDL----FSN
comp32966_c0  124 ------------------------------------------------------------

consensus        361                          .           .            .         


comp26945_c0  130 ------------------------------------------------------------
comp38054_c0   60 TSTAVAVDGLGSTG---------PLL--------------TGTPNHEHSEKNRIHY----
comp36880_c0  145 --VPS-R-----------------L--------------------EGKGEFVEPPETYPG
comp38259_c0  150 --PSV-W-LGG----E-YE---GAR--------------------ADPDEYTIVPPWVPF
comp38889_c0  150 --TSS-M--KG----V-SD---DCR--------------------KKPEEFTVPPRWIDF
comp36259_c0  151 --APK-MIEDG----V-FEADAGTL--------------------IQAEMMQLSPILPVS
comp38694_c0  193 --IPF-LREKG----Y-LT-----V--------------------EEPELETRVPELPPL
comp23425_c0  171 --YRNSPVENG----E-NP-----F--------------------YGPDLDKLVNCVPGA
comp37829_c0  160 --FPD-MVKAG----D-LP-----F--------------------QEADMDKLVTSIPGM
PgUGT71A27    151 --LQTLNDKKQDVT-E-FRNSDTEL--------------------LVPSFANPVPA-EVL
MtUGT71G1     155 --LKNRQI--EEVFDD-SDRDHQLL--------------------NIPGISNQVPS-NVL
SvUGT74M1     148 --LHLESQG-------------KGV--------------------DLEELPSILP----P
comp37573_c0  111 --VGKGN---------------LTA--------------------PVEESTTTVTL-PGL
comp31343_c0  158 --VQQGL---------------LSV--------------------PLAAG-SAVTL-PGS
comp36943_c0  144 --IKNRE-----------------------------------------EG-SDVPL----
BvUGT73C10    163 --LRKNREI----LEN-LKSDKEHF--------------------VVPYFPDRVEF-TRP
BvUGT73C11    163 --LRKNREI----LEN-LKSDKEHF--------------------VVPYFPDRVEF-TRP
BvUGT73C12    163 --LRKNREI----VEN-LKSDKEHF--------------------VVPYFPDRVEF-TRP
BvUGT73C13    163 --LRKNHEI----VEN-LKSDKEHF--------------------VVPYFPDRVEF-TRP
MtUGT73K1     157 --IQSH-------PES-FVSDSGPY--------------------QIHGLPHPLTL-PIK
MtUTG73F3     157 --LKTNNLLKS--QTD-SDSDSSSF--------------------VVPNFPHHITL-CGK
GmUGT73F4     150 --VIS--------HPE-LHSDTGPF--------------------VIPDFPHRVTM-PSR
GmUGT73F2     150 --VIS--------HPE-LHSDTGPF--------------------VIPDFPHRVTM-PSR
comp26042_c0   96 AIRRRVASGRTDS-DSENNNDAE-----------IENVGDEGDEDENPVVKSR-FY----
comp35666_c0   84 AIKKRLILGQGVA-MESE----------------------------KSSKGRL-ML----
comp35395_c0   54 ILVLRGTIGVGNLGGG-SSAEAEALKIKAETDEFIQKIRSDGDPSDLTDQNQEEFM----
comp32485_c0   51 ---------------YSGAGDAP--KY--GDFE---------------AQRHWMEI----
comp27554_c0   90 --KATSLSGKGRK---------------------K-------------------------
comp32214_c0   74 --IASRNEGKGKY---------------------K-------------------------
comp34116_c0    8 P-LQTRKLKSS-S----SS------SI-------NG------------GETLKMTK----
comp34883_c0    1 ------------------------------------------------------------
comp32596_c1  128 I-LQQRSLHLSNH----TA------VL-------VN------------TTVKKMRC----
comp38012_c0  151 K-LQTDVVSPT-K----NS------TQ-------TV------------KKKN--------
comp34587_c0    1 ------------------------------------------------------------
comp21103_c0  232 ------------------------------------------------TTTNSVQM----
comp22969_c0   80 S-LP-PQISPR-E----DV------NN-------NV------------AKPRVVAA----
comp31694_c0  251 S-IQKGSIGKQ-T----GR------GI-------SN------------MAPNSVQI----
comp32596_c0    1 ------------------------------------------------------------
comp30460_c1   18 TAFAATVFKEAPE--YYNSADCP--SV--DEDEYNNTF--------CSDQTVHVAM----
comp30728_c0  239 NILATSVV----------------------------------------------------
comp35926_c0  276 NVIAASVV----------------------------------------------------
comp29055_c0  252 RLFSSGVKKAGSLTKYSKSG--------------NEQF--------ATEISKWVF-----
comp32966_c0  124 -------------------------------------I--------AVFVKKWPE-----

consensus        421   .                                                  .      


comp26945_c0  130 ------------------------------------------------------------
comp38054_c0   93 --ALMCTN---------------------------MNQTQTCPNLPLMD-QDYQSPSSIT
comp36880_c0  165 SSNDKRRPFEMNGTKY-ALTEF--------------------DGTISLVQRVYTSLRDSE
comp38259_c0  178 ENNLRYRIFEIRKVLG-SVLQN--------------------ITGVSDTSRFGVTTNACD
comp38889_c0  177 PSNMAFRTFEILRFFKDALVEN--------------------ESGVSDGYRFGKSVEGCE
comp36259_c0  183 NPAIFVW-----LCMG-GLAAQ--------------------KSMFNLILTFGRVIELAQ
comp38694_c0  220 ----KVKDM--DMFPA-DRDRV--------------------DDIYQLLSLMGYQTKASS
comp23425_c0  199 ESFLRVRDL--PGIRG-PK-KM--------------------RQATEIVET--KISTRAS
comp37829_c0  187 ESYLRCRDL--PSFYR-GP-EV--------------------NDVFQIA----NSPYKPS
PgUGT71A27    186 P-SMYVDKE--GGY--------------------------------DYLFSLFRRCRESK
MtUGT71G1     189 P-DACFNKD--GGY--------------------------------IAYYKLAERFRDTK
SvUGT74M1     169 P-KVIVQKL--PKSFL-AYGDNNSHNNNNNNNNNNNNNNMGLHPLVLWLLKDYGNSVKAD
comp37573_c0  133 P-QLAFQDL--PSTVT-VLDQD--------------------RTSLDMMLGQFSNASQAD
comp31343_c0  179 P-SLGVADF--PTFIS-KYDSF--------------------PSVLSLLLNQFSNLSNAD
comp36943_c0  156 P-ELGLSKM------------P--------------------PAILSLIFTQFSNLDGAD
BvUGT73C10    195 Q-VPLATYV--PGEWH-EIKED--------------------------MVE---ADKTSY
BvUGT73C11    195 Q-VPMATYV--PGEWH-EIKED--------------------------IVE---ADKTSY
BvUGT73C12    195 Q-VPVATYV--PGDWH-EITED--------------------------MVE---ADKTSY
BvUGT73C13    195 Q-VPVATYV--PGDWH-EITGD--------------------------MVE---ADKTSY
MtUGT73K1     186 P-SP---------GFA-RLTES--------------------------LIE---AENDSH
MtUTG73F3     191 P-PK---------VIG-IFMGM--------------------------MLE---TVLKSK
GmUGT73F4     178 P-PK---------MAT-AFMDH--------------------------LLK---IELKSH
GmUGT73F2     178 P-PK---------MAT-AFMDH--------------------------LLK---IELKSH
comp26042_c0  139 ----TCIK--------------------------------LFLWLSVLLLGFEIV-----
comp35666_c0  110 ----RFIR--------------------------------GFLVLSLLVLLVDVV-----
comp35395_c0  109 NPNVTYTLG--P------------------------------------------------
comp32485_c0   73 T--------------------------------------------------LH-------
comp27554_c0  102 ----TYV-----------PL---------------TISPSSFQNG------THLFPIALN
comp32214_c0   86 ----GATVG--L-----PVQ---------------GVNPEGMPPS------LSFYSVSLP
comp34116_c0   33 K-------S--L--LK-PTL--------------------TT---ILMILAFYAFFNAFL
comp34883_c0    1 --------M--L--SK-PLI--------------------SFLIFLLLLIPLYLFIGAE-
comp32596_c1  154 D-------M--P--PK-TVM--------------------TISEMNSLLLRRRA---AAG
comp38012_c0  172 -----------T--ML-APT--------------------TISEMTQILLQNRV---SSR
comp34587_c0    1 ------------------------------------------------------------
comp21103_c0  240 E-------L--P--ST-PVK--------------------SLSDMYLLLLKSHN---SSH
comp22969_c0  104 ----------------------------------------QLPPRVV---VSRQ---HRI
comp31694_c0  276 E----------L--ST-PVK--------------------SLPDMYILLLKSQT---SSL
comp32596_c0    1 ------------------------------------------------------------
comp30460_c1   60 TLDVGYLRG--S--MA-AIL--------------SVLQHSSCPQN----IDFHFVASAST
comp30728_c0  247 ------------------VN--------------STALNSNHPEM----LVFHLVTDEVN
comp35926_c0  284 ------------------VN--------------SAIKNADEPWK----HVFHVVTDKMN
comp29055_c0  285 HE----R-G--H--LK-AVN------------------------------VRHNKVGE--
comp32966_c0  134 MN----RAG--G--LE-RHA--------------L---------------TLHLALAK--

consensus        481                                                 ..          


comp26945_c0  130 -------------------------------------------------TVGLYQNFTEL
comp38054_c0  123 CPDYFSWIHEDLR----PWKDTGITREMVERAKRTANFRLVILNGK--AYVEIYEKS--F
comp36880_c0  204 -ALCFRACNEIEGP----Y------------------------------LDYLE-K--QY
comp38259_c0  217 -IVAVRTDTHFEAE----W------------------------------LELLEDK--LY
comp38889_c0  217 -VILFRTSTRFEPE----W------------------------------LNLLQDT--LY
comp36259_c0  217 -YLICNSFYELEPS----A------------------------------FTM-------I
comp38694_c0  253 -GLILNSCEFLEQS----E------------------------------LESLGNE--LH
comp23425_c0  233 -AWILNTFDALEAP----I------------------------------LSQIFA---HC
comp37829_c0  219 -SFIINTFDNLEAP----I------------------------------LSQMAP---CF
PgUGT71A27    211 -AIIINTFEELEPY----A------------------------------INSL-RMDSMI
MtUGT71G1     214 -GIIVNTFSDLEQS----S------------------------------IDALYDHDEKI
SvUGT74M1     225 -FVLLNSFDKLEEE----A------------------------------I-KWISNI---
comp37573_c0  169 -WLLFNSFDQLESK----V------------------------------L-KVMAEV---
comp31343_c0  215 -FVLVNSFQELEHE----V------------------------------V-EWMSKI---
comp36943_c0  183 -WVLFNSFNKLEEE----V------------------------------I-ENLLKH---
BvUGT73C10    222 -GVIVNTYQELEP-----A------------------------------YANGYKEA-RS
BvUGT73C11    222 -GVIVNTYQELEP-----A------------------------------YANDYKEA-RS
BvUGT73C12    222 -GVIVNTYQELEP-----A------------------------------YANDYKEA-RS
BvUGT73C13    222 -GVIVNTCQELEP-----A------------------------------YANDYKEA-RS
MtUGT73K1     206 -GVIVNSFAELDE-----G------------------------------YTEYYENL-TG
MtUTG73F3     211 -ALIINNFSELDGE----E------------------------------CIQHYEKA-TG
GmUGT73F4     198 -GLIVNSFAELDGE----E------------------------------CIQHYEKS-TG
GmUGT73F2     198 -GLIVNSFAELDGE----E------------------------------CIQHYEKS-TG
comp26042_c0  158 -AYFNGWHFGSPHWQIQYLISSQFGVQS-------------I-------FDYVYSHWVMF
comp35666_c0  129 -AHFKGWHYFESPS---LYIPQTSEIQG-------------W-------LHMLYVAWLSF
comp35395_c0  119 --KISNWNQERKI-----WLDSHPDFPS-------------IVNGKPRT-------L---
comp32485_c0   76 -TPVKEWYRNTSSN-----------------------------------------NL---
comp27554_c0  126 -RPLPP------------GHA---------------------------------LTLEVL
comp32214_c0  114 -KGLSK------------GES---------------------------------LTLDVF
comp34116_c0   58 SPITPSSFSKLDST--LPFDSYSL------------------------------------
comp34883_c0   27 --DLKSHFFPLSQS--LKPSSTQS------------------------------------
comp32596_c1  179 -LLRPQWVSARDKE--LISARSLIENAP-------------LVKNDPELYASLFRNVSMF
comp38012_c0  195 -AMRPRWSSVLDQE--LLFAKSQIEGAP-------------LLKGSAELYPPLYRNVSKF
comp34587_c0    1 ------------------------------------------------------------
comp21103_c0  265 -SMIPKWPSERDME--VFSAKAQIGNPP-------------IMKKDKQLYAPLFRNISTF
comp22969_c0  118 -V-----SLPPNEA--LLYAKKQLEHAP-------------IVTDDPHLYAPLFKNVSTF
comp31694_c0  300 -SAIPRWSSLRDKE--VLSTKSQIENPP-------------IVKTDRQLYAPLFRNVSMF
comp32596_c0    1 ----------------------------------------------------------MF
comp30460_c1   97 DADNL-SRTIRKS---FPYLR-----------------------------------FQVY
comp30728_c0  271 YAPMKAWFSMNT----FRGVTVEVQ------------------------------KFEDF
comp35926_c0  308 LAAMQVWFKMRPP---QKGAHVEVK------------------------------AVEEY
comp29055_c0  303 -AGEPAMYRITDD---LEY------------------------------------FVEIY
comp32966_c0  154 -RG--------HE---LHI------------------------------------FTA--

consensus        541   ..   .  ..                                                


comp26945_c0  141 RV---RPSETPKG-----------------------------YSMTDFRQFLRGAYSLKK
comp38054_c0  175 ----------------QSRDTFTLW---------------------GILQLLKMYPGKLP
comp36880_c0  226 NK--------------PVLKIGAVL----------------------------------P
comp38259_c0  240 RK--------------PVLPVGLLL----------------------------------P
comp38889_c0  240 KK--------------PVLPAGLLP----------------------------------P
comp36259_c0  235 PN------------LHPIGPLIG------------------------------------S
comp38694_c0  276 LQ------------MFPIGPLHKCA----------------------------------P
comp23425_c0  255 PK------------TYTVGPLNLLLLE------R---------------------TGSDK
comp37829_c0  241 PK------------IYTLGPLHALLDLVRSISVS---------------------TSTKS
PgUGT71A27    235 PP------------IYPVGPILNLN---GD------------------------------
MtUGT71G1     239 PP------------IYAVGPLLDLK---GQ------------------------------
SvUGT74M1     246 CS------------VKTIGPTIPSTYLDKQ-------------------------IENDV
comp37573_c0  190 ER------------VRTIGPAVPSIYLDKR-------------------------IQSDK
comp31343_c0  236 WP------------IRTVGPTVPSAYLDNR-------------------------MNDDK
comp36943_c0  204 YP------------IRTIGPTVPSMYLDKR-------------------------LVDDK
BvUGT73C10    245 GK------------AWTIGPVSLCNK-----------------------------VGADK
BvUGT73C11    245 GK------------AWTIGPVSLCNK-----------------------------VGADK
BvUGT73C12    245 GK------------AWTIGPVSLCNK-----------------------------VGADK
BvUGT73C13    245 GK------------AWTIGPVSLCNK-----------------------------VGADK
MtUGT73K1     229 RK------------VWHVGPTSLMVE-----------------------------IPKKK
MtUTG73F3     235 HK------------VWHLGPTSLIRK-----------------------------TAQEK
GmUGT73F4     222 HK------------AWHLGPACLVGK-----------------------------RDQ--
GmUGT73F2     222 HK------------AWHLGPACLVGK-----------------------------RDQ--
comp26042_c0  197 --RVAYLAPPLQFLANVCICLFLVQSVDRL-VL-----------------CLGCFWIRYH
comp35666_c0  165 --RADYIAPTIQGLSTFCVALFIIQSIDRM-VL-----------------CIGCLWIKVK
comp35395_c0  149 --LVTGSPPNPC--DNPIGDHYLLKSIKNKIDYCRLHGIEIVYNMAHLDKELAGYWAKLP
comp32485_c0   91 RYWGLDYPPLTAYQSYAHSLF---------------------------------LNYFHP
comp27554_c0  140 LILTNSLEPFPQQIAQSDSQLVYYRDT-----------------------------ALIF
comp32214_c0  128 AILTHSLQPFPEQITQADVQLVLFQDS-----------------------------AYYL
comp34116_c0   80 --LPTPHTPKLKIYVYDLPPKFTYGVIERY-LTA---RNTNP------------------
comp34883_c0   47 --PCTQFPPPIKVFMYDLPFRFNLAMINHQ-IKD---GVSLD------------ARNF--
comp32596_c1  223 KRSY--------------------------------------------------------
comp38012_c0  239 QRSYELMENMLKVYIYSEGEKPIF------------------------------------
comp34587_c0    1 ------MEQNFKIYIYPDGDPNTF------------------------------------
comp21103_c0  309 LSSYELMERTLKVYSYKEGEQPIF------------------------------------
comp22969_c0  157 KRSYELMESILKVYIYKDGKRPVF------------------------------------
comp31694_c0  344 KRSYELMEQTLKVYTYREGARPIF------------------------------------
comp32596_c0    3 KRSYELMDRGLKVYVYGEGSKPIF------------------------------------
comp30460_c1  118 ---TFDDVPVLGLISTSI--------------------------RAALDCPLNYARNYLA
comp30728_c0  297 SWLNASYVPVLKQLQDSDTKNYYF--SGSN-DG---SRTPIKFRNPKYLSMLNHLRFYIP
comp35926_c0  335 KFLNPSYVPVLRQLESANVQKFYF--ENRA-ENATKDANNIKFRNPKYLSMLNHLRFYLP
comp29055_c0  323 EWSGTNWEPYVAD---DVQIQFYM--MSPY-VLKALST---------DKKGLFHTSFKVP
comp32966_c0  164 AASNASFPTYSLP---NL--HF---------------------------------HLSKP

consensus        601                . ..                                         


comp26945_c0  169 KKAIRLNNQTPRKPKLLIISRKRTR------SFTNEG--DIVEMAKSLGFTVTVAEP---
comp38054_c0  198 D------------------LD----MMFDCVDWPV---IIS-RYFNAKRPNATAPPP---
comp36880_c0  238 R------------PTDTI-LE---KR---WADWLGGFEKGSV------------------
comp38259_c0  252 R------------VEHQV-D--DDPKWVTIKQWLDNKDTRTV------------------
comp38889_c0  252 E------------LNETE-DDKQDENWVRIRDWLDKQEQGTV------------------
comp36259_c0  247 S------------QLATF-WP-EDS---SCLSWLDQQAPKSV------------------
comp38694_c0  290 E------------SHSSL-LE-EDA---SCLSWLDTQNPSSV------------------
comp23425_c0  276 S------------SSNSL-RG-ADR---SCLTWLDSQTSNSV------------------
comp37829_c0  268 S------------SNNSL-SE-VDR---GCMTWLDSQPHKSV------------------
PgUGT71A27    250 -------------GQN---SD----EAAVILGWLDDQPPSSV------------------
MtUGT71G1     254 -------------PNPKLDQA----QHDLILKWLDEQPDKSV------------------
SvUGT74M1     269 D-----------YGFNQY-KP----TNEDCMKWLDTKEANSV------------------
comp37573_c0  213 E-----------HSLNMF-TP----SDVDYIEWLNSKGAGSV------------------
comp31343_c0  259 D-----------YGLNLF-DP----NRATCIDWLNAKETGTV------------------
comp36943_c0  227 D-----------YGYNLY-QS----TGDICTNWLNTKEIGSV------------------
BvUGT73C10    264 A-----------ERGNKA-DI----DQDECLKWLDSKEEGSV------------------
BvUGT73C11    264 A-----------ERGNKA-DI----DQDECLKWLDSKEEGSV------------------
BvUGT73C12    264 A-----------ERGNKA-DI----DQDECLKWLNSKEEGSV------------------
BvUGT73C13    264 A-----------ERGNKA-DI----DQDECLKWLNSKEEGSV------------------
MtUGT73K1     248 KVV---------STENDS-SI----TKHQSLTWLDTKEPSSV------------------
MtUTG73F3     254 S-----------ERGNEG-AV----NVHESLSWLDSERVNSV------------------
GmUGT73F4     239 ------------ERGEKS-VV----SQNECLTWLDPKPTNSV------------------
GmUGT73F2     239 ------------ERGEKS-VV----SQNECLTWLDPKPTNSV------------------
comp26042_c0  237 K-I---------KPIPKQ-AV----R------DIESSEGNAETYF-----------PMV-
comp35666_c0  205 K-I---------KPTIEG-GD----P------FKDDDVEGACYDY-----------PMI-
comp35395_c0  205 --L---------LRRLML-SH----PEVEWIWWMDSDALFTDMVFEIPIA----------
comp32485_c0  118 E-S---------VALFTS-RG----HESSLGKLLMR-------------WTVLSSDVL--
comp27554_c0  171 S-P---------YPI----KQ----QTTYIKT----------PTARLDSYTFLHPTKQVS
comp32214_c0  159 S-P---------YEV----RV----QSLSVGL----------PSKKVESYSKLQNTKLVD
comp34116_c0  116 D-E---------LKRLKY-PG----NQHSAEWYLFSDLISQDRIAGSQIVRALDPNEADL
comp34883_c0   87 P-P---------WPSSSG-LR----QQHSVEYWMMGSLINV--HEGSEAIRVLDPEVADV
comp32596_c1      ------------------------------------------------------------
comp38012_c0  263 --H---------EP--ML-KG----IYASESWFM-E------LMEENKQFVVKDPTKAHM
comp34587_c0   19 --Y---------QTPRKI-TG----KYASEGYFF-Q------NIRES-RFLTTNPDEAHL
comp21103_c0  333 --H---------RP--ET-KG----IYASEGWFM-K------QMEGNKQFSVNDPTKAHL
comp22969_c0  181 --H---------QP--HL-RG----IYASEGWFM-K------LMEENRQFVTRDASRAHL
comp31694_c0  368 --H---------EP--ET-KG----IYASEGWFM-K------QMERNRRFSVNDPRKAHL
comp32596_c0   27 --H---------LP--IL-KG----LYASEGWFM-K------LMEGNKQFTVKDPKKAHL
comp30460_c1  149 N-L---------LPSCVR-RV----V-----YL-DSDLVLVDDIAKLAATPLGDDEKVVL
comp30728_c0  351 E-V---------FPA-LK-KV----V-----FL-DDDVVVQKDITPLFSINLNS---NVN
comp35926_c0  392 E-M---------YPK-LH-QI----L-----FL-DDDVVVQKDLTGLWKIDMNG---KVN
comp29055_c0  368 D-V---------YGVFQF-KV----EYHRLGYT-SLSLAKQI---PVRPFRHNEYERFLT
comp32966_c0  186 ------------------------------------------------------------

consensus        661                                 ..      ..                  


comp26945_c0  218 ------SMGGLSKFAELVNSCDVMMGVHGAGLTNL-----VFL--PTNAILIQ-------
comp38054_c0  229 ----LFRYCGDDK------------------TLDIVFPDWSF------------WGWPEI
comp36880_c0  261 ---VYCAFGSEC-------------------VLEKAQFQELVLGLEMSGFP---------
comp38259_c0  279 ---VYVAFGSEA-------------------VLSQEQTTELALGLELSELP---------
comp38889_c0  281 ---VYVAFGSEA-------------------TLSQAEMTELALGLELSELP---------
comp36259_c0  272 ---IYVAFGSLT-------------------VLNKQQFDELALGLELSGQP---------
comp38694_c0  315 ---IYISFGSFA-------------------AIDETQFREVALGIVQSEQP---------
comp23425_c0  301 ---IYVSFGSLV-------------------QITREQMLEFWYGLVSSGKR---------
comp37829_c0  293 ---IYVSFGSTV-------------------MMTNAQLLEFWYGLVNSGKR---------
PgUGT71A27    272 ---VFLCFGSYG-------------------SFQENQVKEIAMGLERSGHR---------
MtUGT71G1     279 ---VFLCFGSMGV------------------SFGPSQIREIALGLKHSGVR---------
SvUGT74M1     295 ---VYIAFGSVA-------------------RLSVEQMAEIAKALDHSSKS---------
comp37573_c0  239 ---LYASFGSIA-------------------RLGEEQMEELITGIKQSNKH---------
comp31343_c0  285 ---VYASLGSIT-------------------AVTAEQMEELAWGLRDSNYY---------
comp36943_c0  253 ---VYVSFGSAA-------------------KLSAEQMEELAWGLKQSNNF---------
BvUGT73C10    290 ---LYVCLGSIC-------------------SLPLSQLKELGLGLEESQRP---------
BvUGT73C11    290 ---LYVCLGSIC-------------------SLPLSQLKELGLGLEESQRP---------
BvUGT73C12    290 ---LYVCLGSIC-------------------NLPLSQLKELGLGLEESQRP---------
BvUGT73C13    290 ---LYVCLGSIC-------------------NLPLSQLKELGLGLEESQRP---------
MtUGT73K1     276 ---LYISFGSLC-------------------RLSNEQLKEMANGIEASKHQ---------
MtUTG73F3     280 ---LYICFGSIN-------------------YFSDKQLYEMACAIEASGHP---------
GmUGT73F4     264 ---VYVSFGSVC-------------------HFPDKQLYEIACALEQSGKP---------
GmUGT73F2     264 ---VYVSFGSVC-------------------HFPDKQLYEIACALEQSGKS---------
comp26042_c0  264 --LIQIPMCNEKE-----V---------------YQ----------QSIAAVCNLDWPKS
comp35666_c0  232 --LIQIPMCNEKE-----V---------------YE----------QSISAVCQMDWPKD
comp35395_c0  239 ------------------KYDDYNLIVHGYPDLMFEQKSWIAL--NTGSFLFRNCQWSLD
comp32485_c0  148 ---IFFP----------------------------------------------------A
comp27554_c0  203 TELTYGSYYDQ---------PPFSYFP---ILVHFENNHPFSV---LNEFLRE-------
comp32214_c0  191 SEIKYGPYENL---------PAFSYSA---VVVHYENNSPFVV---ARELVRE-------
comp34116_c0  161 ---FYVPFFSS-------LS----LVVNPA----NNKVEERVY--SDEEMQESLMEW---
comp34883_c0  130 ---FFVPFFSS-------TS----FNTHGH----N-MTDPATE--IDRQLQVDLLNF---
comp32596_c1      ------------------------------------------------------------
comp38012_c0  298 ---FYLPFGSF-------RMRLELYDPKHH-------KSLAEF-------LRNYVNI---
comp34587_c0   55 ---FFIPISCH-------KMRG-----KGT----S-YDDMMLI-------VDEYVKM---
comp21103_c0  368 ---FYLPFSTR-------RLEQFLYVPDSH----T-HKNLVAY-------LKSYLDL---
comp22969_c0  216 ---FYLPYSVR-------QLEHALYVPDSH----D-LKPLSIF-------LKDYVNW---
comp31694_c0  403 ---FYLPFSPR-------RLEQFLYVPGSH----S-RRNMVAH-------LKSYLDL---
comp32596_c0   62 ---FYMPFSTR-------MLEHTLYVRNSH----N-RTNLAAY-------LRNYTNI---
comp30460_c1  188 ---AAPEYCNANFT----AYFTPTFWSNPTLSLTFSSRKPCYF--NTGVMVIDLERWRKG
comp30728_c0  386 ---GAVETCMETFH----RYHKYLNYSHPLIRSHFD-PDACGW--AFGMNVFDLVQWRKR
comp35926_c0  427 ---AAVETCFGSFH----RYAQYMNFSHPLIREKFN-PKACAW--AYGMNFFDLDAWRQE
comp29055_c0  409 ---AAFPYYGSSFS----AMAGF-------------------F--VFTVV------Y---
comp32966_c0  186 ------------------TAAGY-------------------L--DQAVI------W---

consensus        721    ... ...                          .  ...     .            


comp26945_c0  258 -IVPWGNLEW---------------------------------------------LCR--
comp38054_c0  255 NIKPWGSLLKELREGNNKITWQNREPYAYWKGNPAVAETRQDL-MKCNVSEKQDWSARVY
comp36880_c0  290 --FLVRLKPPAGAA------------------------------------TVEEALPE--
comp38259_c0  308 --FFWVLRTAVGEN--------------------------------------DCELPA--
comp38889_c0  310 --FFWVWRRPPGAT-------------------------------------EPVTLPT--
comp36259_c0  301 --FLWVVRPDLAVEG----TY---------------------------------AYPD--
comp38694_c0  344 --FLWVIRSGLVARS----SN------------------------SDNEDDLTELLPQ--
comp23425_c0  330 --FLWVLRRDIILGE----DR---------------------------KND----LPA--
comp37829_c0  322 --FLWVMRSNPIVDK----DH---------------------------NNV----ITT--
PgUGT71A27    301 --FLWSLRPSIPKGE----TK-----------------------LQLKYSNLKEILPV--
MtUGT71G1     309 --FLWSNSA------------------------------------------EKKVFPE--
SvUGT74M1     324 --FIWVVRETEKEK-----------------------------------------LPV--
comp37573_c0  268 --FLWVIREKEQSK-----------------------------------------LPD--
comp31343_c0  314 --FLWVVRAREVSK-----------------------------------------LPN--
comp36943_c0  282 --FVWVVRASEEDR-----------------------------------------LPA--
BvUGT73C10    319 --FIWVVRGWEKNKE-----------------------------------LLEWFSES--
BvUGT73C11    319 --FIWVVRGWEKNKE-----------------------------------LLEWFSES--
BvUGT73C12    319 --FIWVIRGWEKNKE-----------------------------------LHEWFSES--
BvUGT73C13    319 --FIWVIRGWEKNKE-----------------------------------LLEWFSES--
MtUGT73K1     305 --FLWVVHGKEGE-------------------------------------DEDNWLPK--
MtUTG73F3     309 --FIWVVPEKKGKED-------------------------------ESEEEKEKWLPK--
GmUGT73F4     293 --FIWIVPEKKGKEY----E-------------------------NESEEEKEKWLPK--
GmUGT73F2     293 --FIWIVPEKKGKEY----E-------------------------NESEEEKEKWLPK--
comp26042_c0  292 NMLI--------QVL----D-----------------DSDDLTTQLMIKEEVTKWHREGA
comp35666_c0  260 RFLV--------QVL----D-----------------DSDDDSIQWLIKGEVSKWSQKGI
comp35395_c0  279 LLDAWAPMGPKGPVR----DEAGKILTAYLKGRPSFEADDQSALIYLLISQKDVWMNKV-
comp32485_c0  153 AI----------------------------------------------------------
comp27554_c0  241 ------------------------------------------------------------
comp32214_c0  229 ------------------------------------------------------------
comp34116_c0  198 -L----------------------------------------------------------
comp34883_c0  166 -L----------------------------------------------------------
comp32596_c1      ------------------------------------------------------------
comp38012_c0  331 -I----------------------------------------------------------
comp34587_c0   85 -L----------------------------------------------------------
comp21103_c0      ------------------------------------------------------------
comp22969_c0  251 -I----------------------------------------------------------
comp31694_c0  438 -I----------------------------------------------------------
comp32596_c0   97 -I----------------------------------------------------------
comp30460_c1  239 DY----------------------------------------------------------
comp30728_c0  436 NV----------------------------------------------------------
comp35926_c0  477 KS----------------------------------------------------------
comp29055_c0  432 ------------------------------------------------------------
comp32966_c0  198 ------------------------------------------------------------

consensus        781     .                                                       


comp26945_c0  270 -----------HYFGEPA---V--GMKL-RYLDYDIREEESTLIEQYPHDHPV-------
comp38054_c0  314 AQDWVD-ENIKGYKGSNL--AD--QCNF-RYKIY-IEGSAWSVSEKYILACDSL------
comp36880_c0  310 -----------GFAERVR--GR--GVVHGGWVQQ-T----------LILAHPS------V
comp38259_c0  326 -----------GFEDGVK--GR--GYICRTWAPQ-L----------KILGHPS------V
comp38889_c0  329 -----------EFEDRVK--GR--GIVWRGWVPQ-L----------KILSHSS------I
comp36259_c0  320 -----------GFEERIG--SR--GHMV-GWAPQ-R----------EVLAHPS------I
comp38694_c0  372 -----------EF-SEMG--AK--GCIV-KWAPQ-Q----------KVLAHPA------V
comp23425_c0  351 -----------KLTEGTK--DR--GYMV-EWSPQ-Q----------EVLLHPA------V
comp37829_c0  343 -----------ELEEGTK--ER--GFIV-GWSPQ-E----------EVLVHPA------V
PgUGT71A27    330 -----------GFLDRTS--CV--GKVI-GWAPQ-V----------AVLAHKA------V
MtUGT71G1     323 -----------GFLEWMELEGK--GMIC-GWAPQ-V----------EVLAHKA------I
SvUGT74M1     339 -----------DLVEKIS--GQ--GMVV-PWAPQ-L----------EVLAHDA------V
comp37573_c0  283 -----------GFAEQVR--DK--GLLL-PWCSQ-L----------EVLAHEA------V
comp31343_c0  329 -----------SFTEYTN--EK--GLVV-SWCPQ-L----------EVLQHPA------L
comp36943_c0  297 -----------AFKEEKL--EK--GLVV-TWSPQ-L----------EVLSHKA------V
BvUGT73C10    340 -----------GFEERVK--DR--GLLIKGWSPQ-M----------LILAHHS------V
BvUGT73C11    340 -----------GFEERVK--DR--GLLIKGWSPQ-M----------LILAHHS------V
BvUGT73C12    340 -----------GFEERIK--DR--GLLIKGWAPQ-M----------LILSHHS------V
BvUGT73C13    340 -----------GFEERIK--DR--GLLIKGWAPQ-M----------LILSHHS------V
MtUGT73K1     324 -----------GFVERMKEEKK--GMLIKGWVPQ-A----------LILDHPS------I
MtUTG73F3     334 -----------GFEERNI-GKK--GLIIRGWAPQ-V----------KILSHPA------V
GmUGT73F4     320 -----------GFEERNR--EK--GMIVKGWAPQ-L----------LILAHPA------V
GmUGT73F2     320 -----------GFEERNR--EK--GMIVKGWAPQ-L----------LILAHPA------V
comp26042_c0  323 NILYRHRVIRDGYKAGNL--KS--AMNC-SYVKD------YEFVA-IFDA----------
comp35666_c0  291 NIIYRHRLVRTGYKAGNL--KS--AMNC-DYVKG------YEFVA-IFDA----------
comp35395_c0  334 --YLENAYYLHGYWAGLV--DRYEEMAD-KYHPG-LGDERWPFVT-HFVGCKPC------
comp32485_c0  155 -------CFVFAYYGDRG--GRG------------KEDMAW--NL-VMILLNPCLILIDH
comp27554_c0  241 --------IVISHWGSVQ--IT---------------DHYK--LV-HAGARHTG------
comp32214_c0  229 --------IEISHWGNVQ--IT---------------EHYN--LV-HSGAENKG------
comp34116_c0  199 --------EGQVYWKRNN--GW---------------DHVF-------ICQDPN------
comp34883_c0  167 --------SKSKYWQRSG--GR---------------DHVI-------PMHHPN------
comp32596_c1      ------------------------------------------------------------
comp38012_c0  332 -------SEKYSSGIGLA--ER---------------IIFS--LL-VMTGHH-N------
comp34587_c0   86 -------SMKYGYWNRTL--GA---------------DHFF------VTCHD-V------
comp21103_c0      ------------------------------------------------------------
comp22969_c0  252 -------AGKYPFWNQTR--GA---------------DHFL------VACHD-W------
comp31694_c0  439 -------SAKYPFWNRTG--GA---------------DHFL------VGCHD-W------
comp32596_c0   98 -------AAKYPFWNRTT--GA---------------DHFF------VACHD-W------
comp30460_c1  241 -------TTKIVEWMELQ--KR---------------MRIY-------------------
comp30728_c0  438 -------TGIYHYWQENN--VD---------------RTLW-------------------
comp35926_c0  479 -------TEQYHYWQNLN--ED---------------RTLW-------------------
comp29055_c0  432 -----------LYNK*--------------------------------------------
comp32966_c0  198 -----------KLFQT--------------------------------------------

consensus        841             .        .  .     .                .. .         


comp26945_c0  306 --------------L----------------------RDPTSIEKQGWNSLKAI------
comp38054_c0  361 ----------TLI------------------------------VTPKYYDIFTR--SLAP
comp36880_c0  338 GCFVG---------------------------------------HCGFGSMWESLVSDCQ
comp38259_c0  354 GGFLT---------------------------------------HSGWSSVIESLAFGCP
comp38889_c0  357 GGSLM---------------------------------------HSGWSSVIESLGFGLP
comp36259_c0  347 ACFLT---------------------------------------HCG-------------
comp38694_c0  398 GGFWT---------------------------------------HNGWNSTLESICEGVP
comp23425_c0  378 GGFLT---------------------------------------QAGWSSILESILAGVP
comp37829_c0  370 GGFLT---------------------------------------HSGWGSILESIIARVP
PgUGT71A27    357 GGFVS---------------------------------------HCGWNSILESVWYDMS
MtUGT71G1     352 GGFVS---------------------------------------HCGWNSILESMWFGVP
SvUGT74M1     366 GCFVS---------------------------------------HCGWNSTIEALSFGVP
comp37573_c0  310 GCFLT---------------------------------------HCGWNSTIEALSMGVP
comp31343_c0  356 GCFLT---------------------------------------HCGWNSTLEALSLGVP
comp36943_c0  324 GCFMT---------------------------------------HCGWNSTVEGVSLGVP
BvUGT73C10    368 GGFLT---------------------------------------HCGWNSTLEGITSGVP
BvUGT73C11    368 GGFLT---------------------------------------HCGWNSTLEGITSGIP
BvUGT73C12    368 GGFLT---------------------------------------HCGWNSTLEGLTAGLP
BvUGT73C13    368 GGFLT---------------------------------------HCGWNSTLEGLTAGLP
MtUGT73K1     354 GGFLT---------------------------------------HCGWNATVEAISSGVP
MtUTG73F3     363 GGFMT---------------------------------------HCGGNSTVEAVSAGVP
GmUGT73F4     348 GGFLS---------------------------------------HCGWNSSLEAVTAGVP
GmUGT73F2     348 GGFLS---------------------------------------HCGWNSSLEAVTAGVP
comp26042_c0  361 -DFQP---------T---------SDFLKKTVPHFKDNEEIALVQARWSFVNKDENLLTR
comp35666_c0  329 -DFQP---------N---------PDFLKLTVPHFKGNPELGLVQARWSFVNKDENLLTR
comp35395_c0  381 -GSY-----GDYPVERCLSSMERAYNFADNQVLQLYGFSHRGLLSPKVKRIRND--TVTP
comp32485_c0  191 GHFQYNCISLGLTIGAV-----------------AAILCKHDVIACSLFSLA--------
comp27554_c0  267 -VFSR----VEYQSRPS-----------------ISGKSSFKHLLVRL----------PP
comp32214_c0  255 -GFSR----LDYQSGSP-----------------IRGMSSFSLLFASL----------PP
comp34116_c0  221 -ALYK----VVDRVKNG-----------------ILLVSDFGRLRPDQASLVKD--VILP
comp34883_c0  189 -AFRF----LRDQVNAS-----------------ILIVADFGRFPKTMSRLSKD--VVAP
comp32596_c1      ------------------------------------------------------------
comp38012_c0  358 -KPSS-----------------------------IWVLAS-GL-SA-ILILLGD--SKLA
comp34587_c0  109 -GVRA-TEGVDYLVKNS-----------------IRVVCSPSY-NVGF-IPHKD--VALP
comp21103_c0      ------------------------------------------------------------
comp22969_c0  275 -GPYT-VTEHEELLRNT-----------------IKALCNSDL-SEGIFVPGRD--VSLP
comp31694_c0  462 -TPHE-TSQ---YMNNC-----------------IRALCNADV-SEGF-AIGKD--VSLP
comp32596_c0  121 -APYE-TRH---HMERC-----------------IKSMCNADV-TQGF-KIGMD--VSFP
comp30460_c1  258 ---------------------------------------ELGSLPPFLLAFAG---NIAP
comp30728_c0  455 ---------------------------------------KLGTLPPGLLTFYG---LTEP
comp35926_c0  496 ---------------------------------------KLGTLPPGLITFYS---TTKP
comp29055_c0      ------------------------------------------------------------
comp32966_c0      ------------------------------------------------------------

consensus        901 ...                                         . .. .        ..


comp26945_c0  324 -----------YLD-----KQNVK------------LDVNRFKG--------TL------
comp38054_c0  379 VHH--------YWPVKDDDKCRSIKF----------------------------------
comp36880_c0  359 LV---------LVP-HFADQFVNTRFMSRGLKVAVEVERRPED---------GWFTKENV
comp38259_c0  375 LV---------LLP-LINDQSIVARSLVW-KKIAFEIPRDEET---------GWFSKESV
comp38889_c0  378 LV---------LMP-IVNEQPLLARLLTW-KNIGMEIPRNDQD---------GSFTGQTV
comp36259_c0      ------------------------------------------------------------
comp38694_c0  419 ML---------CSP-SFGDQKVNARYVSEVWKVGLYLENGL--------------EHEEI
comp23425_c0  399 ML---------CWP-QFGDQLVNSRFMSEVWKIGLDMKDRC--------------DRFTV
comp37829_c0  391 MV---------CFP-FIADQYINSRFVDDVWKVGLDMKDTC--------------DRLTI
PgUGT71A27    378 VA---------TWP-MYGEQQLNAFEMVKELGLAVEIEVDYRN--EY-NKTGFIVRADEI
MtUGT71G1     373 IL---------TWP-IYAEQQLNAFRLVKEWGVGLGLRVDYRK--GS-----DVVAAEEI
SvUGT74M1     387 IL---------AMP-QFLDQLVDAHFVDRVWGVGIAPTVDEN----------DLVTQEEI
comp37573_c0  331 MV---------AMP-HIWDQFTNAKFIEDVWELGVRVKKDEM----------GIVRGKEL
comp31343_c0  377 MV---------ALP-QLSDQPTNAKYVEDVFKVGIRAGVDEN----------GISTRTEI
comp36943_c0  345 MI---------AMP-QFLDQFVDAKFVQDVWEVGVRPKLDES---------KDIVNRYEI
BvUGT73C10    389 LL---------TWP-LFGDQFCNQKLVVQVLKVGVSAGVEEVTNWGEEEKIGVLVDKEGV
BvUGT73C11    389 LL---------TWP-LFGDQFCNQKLVVQVLKVGVSAGVEEVTNWGEEEKIGVLVDKEGV
BvUGT73C12    389 LL---------TWP-LFADQFCNEKLAVQVLKAGVSAGVDQPMKWGEEEKIGVLVDKEGV
BvUGT73C13    389 LL---------TWP-LFADQFCNEKLAVQVLKAGVSAGVDQPMKWGEEEKIGVLVDKEGV
MtUGT73K1     375 MV---------TMP-GFGDQYYNEKLVTEVHRIGVEVGAAEWSMSPYDAK-KTVVRAERI
MtUTG73F3     384 MI---------TWP-VHGDQFYNEKLITQFRGIGVEVGATEWCTSGVAER-KKLVSRDSI
GmUGT73F4     369 MI---------TWP-VMADQFYNEKLITEVRGIGVEVGATEWRLVGYGER-EKLVTRDTI
GmUGT73F2     369 MI---------TWP-VMADQFYNEKLITEVRGIGVEVGATEWRLVGYGER-EKLVTRDTI
comp26042_c0  402 LQ----NINLAFHFEVE--QQVNGIFINF-F--GFNGTAGVWRI-KALEESGGWLERTTV
comp35666_c0  370 LQ----NINLCFHFEVE--QQVNGTFLNF-F--GFNGTAGVWRI-QALEESGGWLERTTV
comp35395_c0  433 LENVD--------------K----------------------------------------
comp32485_c0  226 LNH--KQMSLYFAP----------------------AFFGHLFG-KCIRHQNPILRLSKL
comp27554_c0  295 RVHS-----VYYRDGIGN------------------ISTSHLRL-GA-RKSELEIEP---
comp32214_c0  283 RAHS-----VYYRDEIGN------------------ISTSHLRG-DN-RRTELEIEP---
comp34116_c0  257 YSHR---IN-SFNGDVGVD---ER------------NSLLFFMG-NRYRKEGGKIRDM--
comp34883_c0  225 YVHV---VE-SFDDDSSSDPFASR------------TTLLFFRG-RTIRKDEGIVRAK--
comp32596_c1      ------------------------------------------------------------
comp38012_c0  383 RTYLFQ*-----------------------------------------------------
comp34587_c0  146 QVLQ------PYPLPAGGDDVENR------------TTLGFWAG-HR----NSKIRVR--
comp21103_c0      ------------------------------------------------------------
comp22969_c0  313 ETTIRKPRK-PLRGIGNGKRVSQR------------PILAFFAG-NM----HGRVRPI--
comp31694_c0  496 ETMIRQARN-PLRELG-GKPASER------------QTLAFFAG-GI----HGYLRPI--
comp32596_c0  155 ETYVRSVRN-TVRDIG-GEPASNR------------SILAFFAG-AM----HGYLRPI--
comp30460_c1  276 VDHKWNQ-----------------------------------------------------
comp30728_c0  473 LDPRWHVLGLGYTN-V-DPKVIEK------------GAVLHFNG-NS----KPWLKIGM-
comp35926_c0  514 LDKSWHVLGLGYNPSI-SMDDISR------------AAVIHYNG-NM----KPWLDIAM-
comp29055_c0      ------------------------------------------------------------
comp32966_c0      ------------------------------------------------------------

consensus        961 ..           .    ..             .                    .    .


comp26945_c0  342 ----------------------------------------------------VKALRLLH
comp38054_c0  397 --------AVDWG--------------------------------------------NSH
comp36880_c0  400 CKAV----KSVLDEGS-VIGEEVRVN--------------------------HKKLCQLL
comp38259_c0  415 AESL----RRLVVD---EEGELYRSK--------------------------ALQLKRIV
comp38889_c0  418 ADIL----RKVMVG---AEGEQYRTM--------------------------ARGLRKVF
comp36259_c0      ------------------------------------------------------------
comp38694_c0  455 ARYI----TRLMMKENNKERDEMMIH--------------------------VTDLKEKA
comp23425_c0  435 EKMV----NDLMDH----KSEELRKS--------------------------MDQISELA
comp37829_c0  427 QKMV----IDLMDG----KREELMRS--------------------------MDKISKMA
PgUGT71A27    425 ETKI----KKLMMD-E--KNSEIRKK--------------------------VKEMKEKS
MtUGT71G1     416 EKGL----KDLM---D--KDSIVHKK--------------------------VQEMKEMS
SvUGT74M1     427 SRCL----DEMMGGGP--EGEKIKKN--------------------------VAMWKELT
comp37573_c0  371 ESCI----REVMDG-E--RGETIKAN--------------------------AARWRESA
comp31343_c0  417 VRCL----RQIMEG-E--QGRVVKRK--------------------------ADAWKFLA
comp36943_c0  386 ETCI----REVMEG-E--KSKKIKGN--------------------------SSEWRALA
BvUGT73C10    439 KKAV----EELMGESD--DAKEIRKR--------------------------VKELGQLA
BvUGT73C11    439 KKAV----EELMGESD--DAKERRKR--------------------------VKELGQLA
BvUGT73C12    439 KKAV----EELMGESD--DAKEIRRR--------------------------AKELGELA
BvUGT73C13    439 KKAV----EELMGESD--DAKEIRRR--------------------------AKELGELA
MtUGT73K1     424 EKAV----KKLMDSNG--EGGEIRKR--------------------------AKEMKEKA
MtUTG73F3     433 EKAV----RRLMDGGD--EAENIRLR--------------------------AREFGEKA
GmUGT73F4     418 ETAI----KRLMGGGD--EAQNIRRR--------------------------SEELAEKA
GmUGT73F2     418 ETAI----KRLMGGGD--EAQNIRRR--------------------------SEELAEKA
comp26042_c0  452 E------------------DMDIAVRAH--------------------------------
comp35666_c0  420 E------------------DMDIAVRAH--------------------------------
comp35395_c0  439 --------------------FDIR------------------------------------
comp32485_c0  261 GLVVVGTFVVVWWPYL--YSMKSTTEVLSRLAPFERGIYEDYVANFWCTTSILIKWKKLF
comp27554_c0  327 -------RYPLFGGWK--ATFVIGYGLPLE-----DFVFESSDG----------------
comp32214_c0  315 -------RFPLFGGWR--TAFTIGYGLPLE-----DYLFA-SEG----------------
comp34116_c0  295 -------LFQVLEKEK---DVIIKHGAQSR-----ESRRM-ATK----------------
comp34883_c0  266 -------LAKILKGYQ---DVHYERSVASD-----ATIKA-SSE----------------
comp32596_c1      ------------------------------------------------------------
comp38012_c0      ------------------------------------------------------------
comp34587_c0  181 -------LAAIWENDT---ELDISSNRISR-----AIGPLIYQK----------------
comp21103_c0      ------------------------------------------------------------
comp22969_c0  353 -------LLKHWRGKD--ADMRIYGPLPSR-----VARNMSYAV----------------
comp31694_c0  535 -------LLKYWENKD--PDMKIYGSMGRG-----KKNKANYVQ----------------
comp32596_c0  194 -------LLSYWKDKD--PDMKIYGRMPFG-----VASKMNYIR----------------
comp30460_c1      ------------------------------------------------------------
comp30728_c0  513 -----EKYKPLWDKYV-----DYSNP----------------------------------
comp35926_c0  555 -----NQFRPLWTKYV-----DYEME----------------------------------
comp29055_c0      ------------------------------------------------------------
comp32966_c0      ------------------------------------------------------------

consensus       1021           ..          .                                     


comp26945_c0  350 R*----------------------------------------------------------
comp38054_c0  405 KKKAQEIGKAASNFIHT------NLTMEYVYDYMFHLLNEYSKL----------LKYTPS
comp36880_c0  429 SDEARQ-----------------SSYINNFFAKLQELRN*--------------------
comp38259_c0  442 TDSALQ-----------------DKFIDELDKCLREYRHNKEEQQ---QAFSPN*-----
comp38889_c0  445 SDKIEQ-----------------NQYIDDIISYLKEHHRVNKMSCSGGDKLLS*------
comp36259_c0      ------------------------------------------------------------
comp38694_c0  485 EISLRKEGSS-------------YGSLDNLMEYIFSK*----------------------
comp23425_c0  461 KKSVAEGGSS-------------HSNFEKFVLDIKSMV*---------------------
comp37829_c0  453 IKSVSQGGNS-------------YNNLNTLIEDIKKHV*---------------------
PgUGT71A27    452 RVAMSENGSS-------------YTSLAKLFEKIM-------------------------
MtUGT71G1     441 RNAVVDGGSS-------------LISVGKLIDDITGSN----------------------
SvUGT74M1     455 KEALDKGGSS-------------DKHIDEIIEWLSSS-----------------------
comp37573_c0  398 REAVDAGGSS-------------ELNIDEFVQSLNMELASRESVH*--------------
comp31343_c0  444 KKAVDNGGSS-------------DKNIEDFASNLCLKLNFN*------------------
comp36943_c0  413 IEAVDVGGSS-------------DRNIDEIVATLL---KF*-------------------
BvUGT73C10    467 HKAVEEGGSS-------------HSNITSLLEDIMQLAQPNN------------------
BvUGT73C11    467 QKAVEEGGSS-------------HSNITSLLEDIMQLAQSNN------------------
BvUGT73C12    467 HKAVEEGGSS-------------HSNITSLLEDIMQLAQSNN------------------
BvUGT73C13    467 HKAVEEGGSS-------------HSNITSLLEDIMQLAQSNN------------------
MtUGT73K1     452 WKAVQEGGSS-------------QNCLTKLVDYLHSVVVTKSVELN--------------
MtUTG73F3     461 IQAIQEGGSS-------------YNNLLALIDELKRSRDLKRLRDLKLDD----------
GmUGT73F4     446 KQSLQEGGSS-------------HNRLTTLIADLMRLRDSKSAT----------------
GmUGT73F2     446 KQSLQEGGSS-------------HNRLTTLIADLMRLRDSKSAT----------------
comp26042_c0  462 -----------------------------LHGWKFVYLNDVECQCELPESYEAYRKQQHR
comp35666_c0  430 -----------------------------LNGWKFIYLNDVKVLCEVPESYEAYRKQQHR
comp35395_c0  443 ---------------------------------------------------------RPL
comp32485_c0  319 STQTLKLFSLSATLLSLLSSMLQQIRVPSNKGFLYAMLNSSFAFYLFSFQVHEKSILLPL
comp27554_c0  357 -----------------------------------RRYLNFSFGCPLADTVVEKLTIKVV
comp32214_c0  344 -----------------------------------KRFLNITFGCPMQEMVIDNLIVKVV
comp34116_c0  323 -----------------------------------GMHSSKFCLHPAGDTPSACRLFDAI
comp34883_c0  294 -----------------------------------GMRTSKFCLHPAGDTPSSCRLFDAI
comp32596_c1      ------------------------------------------------------------
comp38012_c0      ------------------------------------------------------------
comp34587_c0  210 -----------------------------------KYYKTKFCICPGGSQVNSARIAESI
comp21103_c0      ------------------------------------------------------------
comp22969_c0  383 -----------------------------------HMKSSKFCICPMGFEVNSPRIVEAI
comp31694_c0  565 -----------------------------------NMKSSKYCICAKGFEVNSPRVVEAI
comp32596_c0  224 -----------------------------------HMQTSKYCICAKGYEVNSPRVVEAI
comp30460_c1      ------------------------------------------------------------
comp30728_c0  529 ----------------------------------------MLQLCNVH*-----------
comp35926_c0  571 ----------------------------------------FVQMCNFGL*----------
comp29055_c0      ------------------------------------------------------------
comp32966_c0      ------------------------------------------------------------

consensus       1081                                  .                          


comp26945_c0      ------------------------------------------------------------
comp38054_c0  449 KPAKAVEFCSE----------MMACHADGLEKKFM----------------LDSTVKSPS
comp36880_c0      ------------------------------------------------------------
comp38259_c0      ------------------------------------------------------------
comp38889_c0      ------------------------------------------------------------
comp36259_c0      ------------------------------------------------------------
comp38694_c0      ------------------------------------------------------------
comp23425_c0      ------------------------------------------------------------
comp37829_c0      ------------------------------------------------------------
PgUGT71A27        ------------------------------------------------------------
MtUGT71G1         ------------------------------------------------------------
SvUGT74M1         ------------------------------------------------------------
comp37573_c0      ------------------------------------------------------------
comp31343_c0      ------------------------------------------------------------
comp36943_c0      ------------------------------------------------------------
BvUGT73C10        ------------------------------------------------------------
BvUGT73C11        ------------------------------------------------------------
BvUGT73C12        ------------------------------------------------------------
BvUGT73C13        ------------------------------------------------------------
MtUGT73K1         ------------------------------------------------------------
MtUTG73F3         ------------------------------------------------------------
GmUGT73F4         ------------------------------------------------------------
GmUGT73F2         ------------------------------------------------------------
comp26042_c0  493 WHSG------PM-------QLFRLCLPDIIRAKISMGKKVNLIFLFF----LLRKLVLPF
comp35666_c0  461 WHSG------PM-------QLFRLCLPAIITSKISFMKKANLILLFF----LLRKLILPF
comp35395_c0  446 LKDNSHS*----------------------------------------------------
comp32485_c0  379 LPASLLALEEPLLFKWFTYFSLFSMYPLLCRDQLILPYIALV-FLFFLVY-HSRNAKGPW
comp27554_c0  382 LPEGSK--------------NATAVGPFTVEQHMETSYSY-LDVVGRPVVVLEKKNVVPD
comp32214_c0  369 LPEGSA--------------DVSVSVPFSVKQGQEVKYSH-LDIVGRPVVVLERNNVVPE
comp34116_c0  348 V---------------------SLCIPVIVSDFVELPFEDVIDYNKIAVFISTNTAVNPG
comp34883_c0  319 V---------------------SHCVPVIVSDKIELPYEDEIDYAEFSLFFSVKEALQPG
comp32596_c1      ------------------------------------------------------------
comp38012_c0      ------------------------------------------------------------
comp34587_c0  235 H---------------------YGCVPVILSDYYDLPFNDILNWRKFAVILKESDVYQL-
comp21103_c0      ------------------------------------------------------------
comp22969_c0  408 Y---------------------HECVPVIIADNFVLPFGDVLDWNSFSVVVAEKDIPNL-
comp31694_c0  590 F---------------------YECVPVIISDNFVPPFFEVLNWEAFAIFVPEKDIPNL-
comp32596_c0  249 F---------------------YECVPVIISDNFVPPFFDVLNWEAFSVIVAEKDIPNL-
comp30460_c1      ------------------------------------------------------------
comp30728_c0      ------------------------------------------------------------
comp35926_c0      ------------------------------------------------------------
comp29055_c0      ------------------------------------------------------------
comp32966_c0      ------------------------------------------------------------

consensus       1141                                                             


comp26945_c0      ------------------------------------------------------------
comp38054_c0  483 --DTMPCN------------------------------------LPPPMDPYTRKF----
comp36880_c0      ------------------------------------------------------------
comp38259_c0      ------------------------------------------------------------
comp38889_c0      ------------------------------------------------------------
comp36259_c0      ------------------------------------------------------------
comp38694_c0      ------------------------------------------------------------
comp23425_c0      ------------------------------------------------------------
comp37829_c0      ------------------------------------------------------------
PgUGT71A27        ------------------------------------------------------------
MtUGT71G1         ------------------------------------------------------------
SvUGT74M1         ------------------------------------------------------------
comp37573_c0      ------------------------------------------------------------
comp31343_c0      ------------------------------------------------------------
comp36943_c0      ------------------------------------------------------------
BvUGT73C10        ------------------------------------------------------------
BvUGT73C11        ------------------------------------------------------------
BvUGT73C12        ------------------------------------------------------------
BvUGT73C13        ------------------------------------------------------------
MtUGT73K1         ------------------------------------------------------------
MtUTG73F3         ------------------------------------------------------------
GmUGT73F4         ------------------------------------------------------------
GmUGT73F2         ------------------------------------------------------------
comp26042_c0  536 YSFTLFCIILPMTMFIPEAELPAWVVCYIPATM------SFLNILPAPK-----------
comp35666_c0  504 YSFTLFCIILPLTMFVPEAELPIWVICYIPVFM------SFLNILPAPR-----------
comp35395_c0      ------------------------------------------------------------
comp32485_c0  437 -RHN--------------GVTNGWGRVMTALLLLCASMHVVYLIVKPPE-KYPYLFDAMI
comp27554_c0  427 --------------------------H-------NTHFQVYYKFKPMLMLAEPLMLALVF
comp32214_c0  414 --------------------------H-------NLHFQVYYRFSNLSLLREPLMLVVGF
comp34116_c0  387 YIVK--------------KLRKISAERILEYQRELKEVKHYFEYDDPN----GTVKEIWR
comp34883_c0  358 YMVE--------------QLKKIERDRWIEMWKKLKNISHHYEFQYPPK-REDAVNMLWR
comp32596_c1      ------------------------------------------------------------
comp38012_c0      ------------------------------------------------------------
comp34587_c0  273 --KD--------------RLKAISNEEFIALHRNLIKVQKHYFWNTPPV-RYDAFHMVMY
comp21103_c0      ------------------------------------------------------------
comp22969_c0  446 --KD--------------ILMAIPLRRYLWMQTNVKMLQKHFLWNPRPV-----------
comp31694_c0  628 --KN--------------ILLSISAEKYLKLQLNVKRVQQHFLWHTKPA-KYDIFHMILH
comp32596_c0  287 --KN--------------ILLSIPYKKYVAMQLAVKKVQRHFIWHSKPV-KYDLFHMTLH
comp30460_c1      ------------------------------------------------------------
comp30728_c0      ------------------------------------------------------------
comp35926_c0      ------------------------------------------------------------
comp29055_c0      ------------------------------------------------------------
comp32966_c0      ------------------------------------------------------------

consensus       1201                                                             


comp26945_c0      ------------------------------------------------------------
comp38054_c0  501 ---------------SARRKTNTIAQVE-TWQNKYWDGKNKQ------------------
comp36880_c0      ------------------------------------------------------------
comp38259_c0      ------------------------------------------------------------
comp38889_c0      ------------------------------------------------------------
comp36259_c0      ------------------------------------------------------------
comp38694_c0      ------------------------------------------------------------
comp23425_c0      ------------------------------------------------------------
comp37829_c0      ------------------------------------------------------------
PgUGT71A27        ------------------------------------------------------------
MtUGT71G1         ------------------------------------------------------------
SvUGT74M1         ------------------------------------------------------------
comp37573_c0      ------------------------------------------------------------
comp31343_c0      ------------------------------------------------------------
comp36943_c0      ------------------------------------------------------------
BvUGT73C10        ------------------------------------------------------------
BvUGT73C11        ------------------------------------------------------------
BvUGT73C12        ------------------------------------------------------------
BvUGT73C13        ------------------------------------------------------------
MtUGT73K1         ------------------------------------------------------------
MtUTG73F3         ------------------------------------------------------------
GmUGT73F4         ------------------------------------------------------------
GmUGT73F2         ------------------------------------------------------------
comp26042_c0  579 SFPFIVPYLLFENTMSVTKFGAMISGLFQLGSAYEWVVTK--------------------
comp35666_c0  547 SFPFIVPYLLFENTMSVTKFNAMVSGLFQLGSSYEWIVTK--------------------
comp35395_c0      ------------------------------------------------------------
comp32485_c0  481 --MFICCSQFVFIA--------FYTNYK------QWK-LD----------CST-------
comp27554_c0  454 FLFFVASVAYLHVDLSIRKS*---------------------------------------
comp32214_c0  441 LFLFITCIVYMHTDLSISKSSASYLAKL------QWDEVQATIQQVQNIINRCLGVHEKL
comp34116_c0  429 Q----VSLKLPHVKLMINRDKRLVS--R------ELSEPD----------CSCLCS----
comp34883_c0  403 Q----VRNKLPETKLSVNRNRRLKI--P------DWWDRR----------RR*-------
comp32596_c1      ------------------------------------------------------------
comp38012_c0      ------------------------------------------------------------
comp34587_c0  316 ELWLRRHVIKY*------------------------------------------------
comp21103_c0      ------------------------------------------------------------
comp22969_c0      ------------------------------------------------------------
comp31694_c0  671 SIWYNRVYQIKAA*----------------------------------------------
comp32596_c0  330 SMWYSRVFTIKADKLAMKNS*---------------------------------------
comp30460_c1      ------------------------------------------------------------
comp30728_c0      ------------------------------------------------------------
comp35926_c0      ------------------------------------------------------------
comp29055_c0      ------------------------------------------------------------
comp32966_c0      ------------------------------------------------------------

consensus       1261                                                             


comp26945_c0      ------------------------------------------------------------
comp38054_c0  527 ------------------------------------------------P*----------
comp36880_c0      ------------------------------------------------------------
comp38259_c0      ------------------------------------------------------------
comp38889_c0      ------------------------------------------------------------
comp36259_c0      ------------------------------------------------------------
comp38694_c0      ------------------------------------------------------------
comp23425_c0      ------------------------------------------------------------
comp37829_c0      ------------------------------------------------------------
PgUGT71A27        ------------------------------------------------------------
MtUGT71G1         ------------------------------------------------------------
SvUGT74M1         ------------------------------------------------------------
comp37573_c0      ------------------------------------------------------------
comp31343_c0      ------------------------------------------------------------
comp36943_c0      ------------------------------------------------------------
BvUGT73C10        ------------------------------------------------------------
BvUGT73C11        ------------------------------------------------------------
BvUGT73C12        ------------------------------------------------------------
BvUGT73C13        ------------------------------------------------------------
MtUGT73K1         ------------------------------------------------------------
MtUTG73F3         ------------------------------------------------------------
GmUGT73F4         ------------------------------------------------------------
GmUGT73F2         ------------------------------------------------------------
comp26042_c0  619 ----------------KSGRSSEGDLVHMVEKDPKHNRANSL------PD---LDAM---
comp35666_c0  587 ----------------KAGRASESDLLAAEERETKALSHAQLHRGSSESELSLLNKMK--
comp35395_c0      ------------------------------------------------------------
comp32485_c0  507 ------------------------------------------------------------
comp27554_c0      ------------------------------------------------------------
comp32214_c0  495 DASLRDLSRTGDIQACKAARKTSDSLLKELAKDL-------------RPSLVFLQSCPQA
comp34116_c0  463 -------NKTGPISTV*-------------------------------------------
comp34883_c0      ------------------------------------------------------------
comp32596_c1      ------------------------------------------------------------
comp38012_c0      ------------------------------------------------------------
comp34587_c0      ------------------------------------------------------------
comp21103_c0      ------------------------------------------------------------
comp22969_c0      ------------------------------------------------------------
comp31694_c0      ------------------------------------------------------------
comp32596_c0      ------------------------------------------------------------
comp30460_c1      ------------------------------------------------------------
comp30728_c0      ------------------------------------------------------------
comp35926_c0      ------------------------------------------------------------
comp29055_c0      ------------------------------------------------------------
comp32966_c0      ------------------------------------------------------------

consensus       1321                                                             


comp26945_c0      ------------------------------------------------------------
comp38054_c0      ------------------------------------------------------------
comp36880_c0      ------------------------------------------------------------
comp38259_c0      ------------------------------------------------------------
comp38889_c0      ------------------------------------------------------------
comp36259_c0      ------------------------------------------------------------
comp38694_c0      ------------------------------------------------------------
comp23425_c0      ------------------------------------------------------------
comp37829_c0      ------------------------------------------------------------
PgUGT71A27        ------------------------------------------------------------
MtUGT71G1         ------------------------------------------------------------
SvUGT74M1         ------------------------------------------------------------
comp37573_c0      ------------------------------------------------------------
comp31343_c0      ------------------------------------------------------------
comp36943_c0      ------------------------------------------------------------
BvUGT73C10        ------------------------------------------------------------
BvUGT73C11        ------------------------------------------------------------
BvUGT73C12        ------------------------------------------------------------
BvUGT73C13        ------------------------------------------------------------
MtUGT73K1         ------------------------------------------------------------
MtUTG73F3         ------------------------------------------------------------
GmUGT73F4         ------------------------------------------------------------
GmUGT73F2         ------------------------------------------------------------
comp26042_c0  651 -----EKEQLISKRKQRHNRMYRKELALAFLLLTASARSLLSAQGIHF-YFLLFQG----
comp35666_c0  629 ----AQQEAATPVPKKRVNKIFRKELALAFLLLTAAVRSLLSAQGVHF-YFLLFQG----
comp35395_c0      ------------------------------------------------------------
comp32485_c0  507 ---------STVKEKKVQ*-----------------------------------------
comp27554_c0      ------------------------------------------------------------
comp32214_c0  542 AQIWPKVEELVVKEKEIEEKLMLKHIT-----VVDSYEKKSGARDAENKVAALQQKLTTL
comp34116_c0      ------------------------------------------------------------
comp34883_c0      ------------------------------------------------------------
comp32596_c1      ------------------------------------------------------------
comp38012_c0      ------------------------------------------------------------
comp34587_c0      ------------------------------------------------------------
comp21103_c0      ------------------------------------------------------------
comp22969_c0      ------------------------------------------------------------
comp31694_c0      ------------------------------------------------------------
comp32596_c0      ------------------------------------------------------------
comp30460_c1      ------------------------------------------------------------
comp30728_c0      ------------------------------------------------------------
comp35926_c0      ------------------------------------------------------------
comp29055_c0      ------------------------------------------------------------
comp32966_c0      ------------------------------------------------------------

consensus       1381                                                             


comp26945_c0      --------------------
comp38054_c0      --------------------
comp36880_c0      --------------------
comp38259_c0      --------------------
comp38889_c0      --------------------
comp36259_c0      --------------------
comp38694_c0      --------------------
comp23425_c0      --------------------
comp37829_c0      --------------------
PgUGT71A27        --------------------
MtUGT71G1         --------------------
SvUGT74M1         --------------------
comp37573_c0      --------------------
comp31343_c0      --------------------
comp36943_c0      --------------------
BvUGT73C10        --------------------
BvUGT73C11        --------------------
BvUGT73C12        --------------------
BvUGT73C13        --------------------
MtUGT73K1         --------------------
MtUTG73F3         --------------------
GmUGT73F4         --------------------
GmUGT73F2         --------------------
comp26042_c0  701 ---VSFLLVGLDLIGEQVQ*
comp35666_c0  680 ---VSFLLVGLDLIGEQMS*
comp35395_c0      --------------------
comp32485_c0      --------------------
comp27554_c0      --------------------
comp32214_c0  597 RHELNELLEFIDEI*-----
comp34116_c0      --------------------
comp34883_c0      --------------------
comp32596_c1      --------------------
comp38012_c0      --------------------
comp34587_c0      --------------------
comp21103_c0      --------------------
comp22969_c0      --------------------
comp31694_c0      --------------------
comp32596_c0      --------------------
comp30460_c1      --------------------
comp30728_c0      --------------------
comp35926_c0      --------------------
comp29055_c0      --------------------
comp32966_c0      --------------------

consensus       1441                     
