# Supplementary material for: Comprehensive Analysis of the Triterpenoid Saponins Biosynthetic Pathway in Anemone flaccida by Transcriptome and Proteome Profiling
Source: Front Plant Sci. 2016 Jul 25;7:1094. doi: 10.3389/fpls.2016.01094 (PMC4958654; doi:10.3389/fpls.2016.01094)
Supplement: FIGURE S1 — Multiple alignment of deduced amino acid sequences of UGTs from Anemone flaccida and other species (representative kinds only). Black shaded and gray shaded boxes show identical and similar amino acids, respectively. The conserved PSPG motif at the C-termini is indicated by the green box. [file Data_Sheet_1.ZIP › supplement/Figure Supplementary 2.docx]

**Group Ⅳ**

**Group Ⅰ**

**Group Ⅴ**

**Group Ⅲ**

**Group Ⅷ**

**Group Ⅹ**

**Group Ⅶ**

**Group Ⅺ**

**Group Ⅸ**

**Group Ⅵ**

**Group Ⅱ**

**Group Ⅻ**
